# Interactive effects of changes in UV radiation and climate on terrestrial ecosystems, biogeochemical cycles, and feedbacks to the climate system

**DOI:** 10.1007/s43630-023-00376-7

**Published:** 2023-02-01

**Authors:** P. W. Barnes, T. M. Robson, R. G. Zepp, J. F. Bornman, M. A. K. Jansen, R. Ossola, Q.-W. Wang, S. A. Robinson, B. Foereid, A. R. Klekociuk, J. Martinez-Abaigar, W.-C. Hou, R. Mackenzie, N. D. Paul

**Affiliations:** 1 Biological Sciences and Environment Program, Loyola University New Orleans, New Orleans, USA; 2 Organismal & Evolutionary Biology (OEB), Faculty of Biological and Environmental Sciences, Viikki Plant Sciences Centre (ViPS), University of Helsinki, Helsinki, Finland; 3 National School of Forestry, University of Cumbria, Ambleside, UK; 4 ORD/CEMM, US Environmental Protection Agency, Athens, GA USA; 5 Food Futures Institute, Murdoch University, Perth, Australia; 6 BEES, University College Cork, Cork, Ireland; 7 Atmospheric Chemistry Observations and Modeling Laboratory, National Center for Atmospheric Research, Boulder, USA; 8 Institute of Applied Ecology, Chinese Academy of Sciences (CAS), Shenyang, China; 9 Global Challenges Program & School of Earth, Atmospheric and Life Sciences, Securing Antarctica’s Environmental Future, University of Wollongong, Wollongong, Australia; 10 Environment and Natural Resources, Norwegian Institute of Bioeconomy Research, Ås, Norway; 11 Antarctic Climate Program, Australian Antarctic Division, Kingston, Australia; 12 Faculty of Science and Technology, University of La Rioja, Logroño (La Rioja), Spain; 13 Department of Environmental Engineering, National Cheng Kung University, Tainan City, Taiwan; 14 Cape Horn International Center (CHIC), Puerto Williams, Chile; 15 Millennium Institute Biodiversity of Antarctic and Subantarctic Ecosystems (BASE), Santiago, Chile; 16 Lancaster Environment Centre, Lancaster University, Lancaster, UK

## Abstract

Terrestrial organisms and ecosystems are being exposed to new and rapidly changing combinations of solar UV radiation and other environmental factors because of ongoing changes in stratospheric ozone and climate. In this Quadrennial Assessment, we examine the interactive effects of changes in stratospheric ozone, UV radiation and climate on terrestrial ecosystems and biogeochemical cycles in the context of the Montreal Protocol. We specifically assess effects on terrestrial organisms, agriculture and food supply, biodiversity, ecosystem services and feedbacks to the climate system. Emphasis is placed on the role of extreme climate events in altering the exposure to UV radiation of organisms and ecosystems and the potential effects on biodiversity. We also address the responses of plants to increased temporal variability in solar UV radiation, the interactive effects of UV radiation and other climate change factors (e.g. drought, temperature) on crops, and the role of UV radiation in driving the breakdown of organic matter from dead plant material (i.e. litter) and biocides (pesticides and herbicides). Our assessment indicates that UV radiation and climate interact in various ways to affect the structure and function of terrestrial ecosystems, and that by protecting the ozone layer, the Montreal Protocol continues to play a vital role in maintaining healthy, diverse ecosystems on land that sustain life on Earth. Furthermore, the Montreal Protocol and its Kigali Amendment are mitigating some of the negative environmental consequences of climate change by limiting the emissions of greenhouse gases and protecting the carbon sequestration potential of vegetation and the terrestrial carbon pool.

**Graphical abstract:**

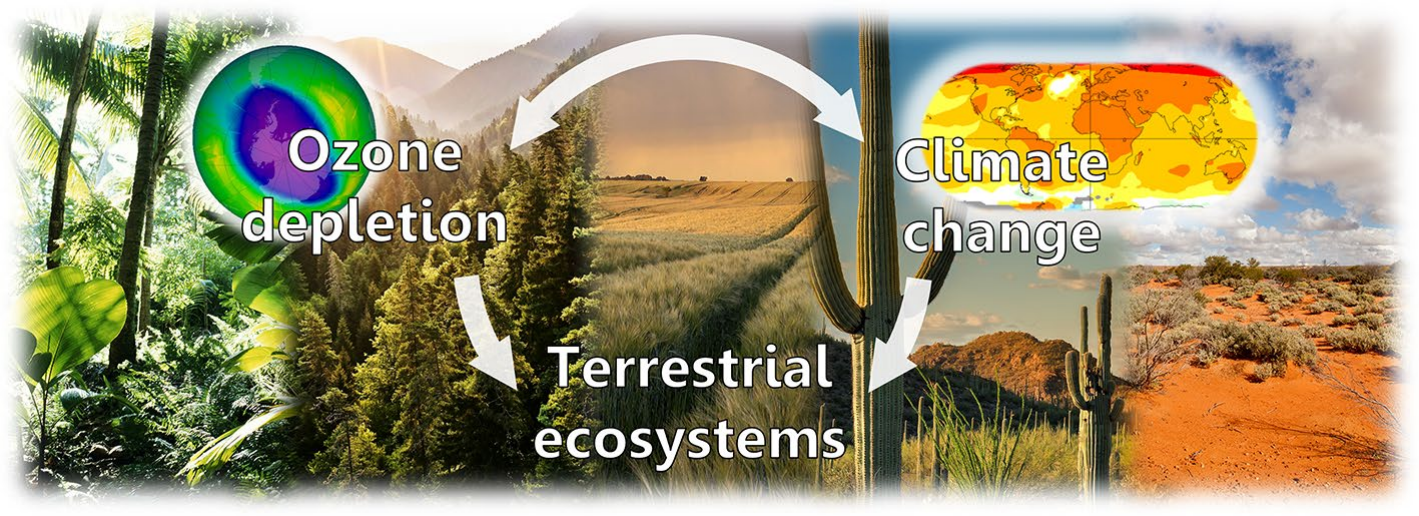

## Introduction

The Montreal Protocol and its Amendments have been highly effective in protecting the Earth’s stratospheric ozone layer and preventing global-scale increases in solar ultraviolet-B radiation (UV-B; wavelengths between 280 and 315 nm) at the Earth’s surface [1]. Consequently, this multilateral treaty, ratified by all 198 United Nations member states, has prevented large-scale detrimental effects of elevated UV-B radiation on agricultural productivity, terrestrial organisms and ecosystems [2–4]. Moreover, because many of the ozone-depleting compounds regulated by the Montreal Protocol are also potent greenhouse gases, this treaty and its Kigali Amendment are playing an important role in mitigating global warming and other environmental effects of climate change [5, 6].

Changes in stratospheric ozone and climate are not independent of one another [7, 8] and both can affect surface ultraviolet radiation (UV; 280–400 nm), especially UV-B radiation [9–11]. According to current projections, which assume full compliance with the Montreal Protocol, future changes in UV radiation reaching the Earth’s surface are likely to be due primarily to changes in climate (i.e. mainly cloud cover, aerosols and surface reflectivity) rather than changes in stratospheric ozone [10, 12, 13]. However, future changes in UV radiation at the Earth’s surface are uncertain: a new study projects an increase in the UV Index of 3–8% over the tropics and mid-latitudes, respectively, by 2100 depending on the greenhouse gas (GHG) scenario used in the model simulations, cloud cover, and aerosol concentrations [10, 14]. Changes in the exposure of organisms and ecosystems to UV radiation also results from increased incidence and extent of wildfires, which generate aerosols (also causing further damage to the ozone layer), and from alterations in vegetation cover from land-use practices (e.g. deforestation), melting of snow and ice, and shifting distribution ranges of species responding to climate change [10, 12, 15–18] (Summarised in Table 1). In this assessment, we address how the expected, rather small changes in UV irradiation interact with the ongoing changes in climate to affect food security, biodiversity, biogeochemical cycles and feedbacks to the climate system.

**Table 1:** Summary of the effects of various climate change-driven factors on the potential exposure of terrestrial plants and animals to UV radiation

Climate change effect	Effect on exposure to UV radiation
Migration or range shift to higher elevations	** + **
Migration or range shift to higher latitudes	**- -**
Altered phenology (seasonal development)	**-/ + **
Deforestation (wet regions)	** + + + **
Shrub encroachment (dry regions)	**- - -**
Altered cloud cover	**- - -/ + + + **
Change in aerosols	**- -/ + + **
Decreased snow/ice cover	**- / + + **

Effects show direction (i.e. decreases (−) or increases ( +)) in exposure to UV radiation with the relative magnitude of these changes indicated by the number of negative and positive signs. In some cases (e.g. altered phenology), changes may either increase or decrease UV exposure depending on the circumstances and species. Changes in exposure to UV radiation resulting from modifications in land cover (i.e. deforestation and shrub encroachment) refer to effects on ground-dwelling, understorey organisms. The effects on exposure to UV radiation shown here do not include changes in stratospheric ozone. Additional information and relevant references are provided in the text that follows

Since our last Quadrennial Assessment [12, 19], the Earth’s climate has continued to change and the frequency and intensity of extreme climate events (e.g. heat waves, droughts, and storms), and those events resulting from a combination of weather extremes and other drivers (e.g. wildfires), have increased [20, 21]. As global warming and its consequences continue to increase, there is renewed interest in possible technological interventions to reduce the warming. Stratospheric Aerosol Injection (SAI), an intervention that involves Solar Radiation Management (SRM), has received the most attention due to its potential feasibility. SAI would involve injecting reflective aerosols, such as sulphate, into the stratosphere to reflect incoming solar radiation away from the Earth’s surface [22]. There are many uncertainties associated with this intervention, including risks to the stratospheric ozone layer that could increase ground-level UV irradiance [23–25]. In addition to the risks associated with the initiation of SAI, once adopted, any subsequent termination of this climate intervention would lead to a rapid increase in temperature and extreme deleterious effects on ecosystems [26, 27]. This, and other SRM interventions, would likely expose the Earth’s ecosystems to new and potentially rapidly changing combinations of UV radiation and other biotic and abiotic environmental factors [28].

In this Quadrennial Assessment, we evaluate the current state of the science on the changes in stratospheric ozone, solar UV radiation and their interactions with climate change as they affect terrestrial ecosystems and biogeochemical cycles in the context of the Montreal Protocol [29]. We also address key gaps in knowledge and how these interacting effects and the Montreal Protocol will have a bearing on the targets of the United Nations Sustainable Development Goals (SDGs) and their targets.

## Effects of stratospheric ozone depletion on climate and extreme climate events on exposure to UV radiation

While both stratospheric ozone depletion and climate change can modify the amount of UV radiation reaching terrestrial ecosystems [8, 10], ozone depletion itself can also contribute to climate change by modifying atmospheric circulation patterns and altering regional patterns of wind, precipitation and temperature [30–32]. The impacts of these changes in climate on terrestrial ecosystems have been most pronounced in Antarctica and in the high latitudes of the Southern Hemisphere, although there is evidence of ozone-driven climate change in Arctic regions as well [33]. In addition to the effects of climate change on UV irradiation outlined above (Table 1), extreme events linked to climate change (e.g. droughts, floods, heat waves, fires) may abruptly change UV radiation conditions for many organisms. Below, we assess recent findings on the effects of ozone-driven climate change on polar ecosystems and the potential effects of extreme events on the exposure of terrestrial ecosystems in general to UV radiation.

### Recent stratospheric ozone depletion and climate change effects on polar ecosystems

The impact of stratospheric ozone depletion on polar ecosystems is a complex interplay between the consequences of changing surface UV radiation, and effects caused by shifts in the weather and climate due to the associated cooling of the lower stratosphere [12, 29, 34]. The increased UV irradiance in the polar regions as a direct result of ozone depletion has been documented since the late 1970s (ozone hole era) [8]. This has particularly been the case in the Antarctic region, where measurements show that the UV Index at the surface in late spring and early summer has, at times, been similar to that at mid- and subtropical latitudes [10, 35]. In the past, it was assumed that snow and ice cover would provide plants and surface organisms some protection from the high UV irradiances that occur during the peak of ozone depletion, but with climate warming accelerating the melting of snow and sea ice, Antarctic organisms are increasingly being exposed to this elevated UV radiation. How these high UV irradiances in late springtime impact the resident plants and animals is not entirely clear; studies conducted at the end of the twentieth century found relatively small effects on plants exposed to the elevated UV radiation experienced at that time. This was likely due to the inherent adaptations, UV-protective mechanisms and acclimation responses of these species in order to survive extreme environments [36–38]. Without the Montreal Protocol, the maximum UV Index would have potentially increased from pre-ozone depletion levels of 6–20, exposing coastal Antarctic organisms to UV Indices at the end of this century that would be greater than those experienced today in the tropics [10]. These extreme UV radiation conditions would likely have exceeded the UV-tolerances of many Antarctic organisms. In the Arctic, surface UV-B irradiance has also been elevated in recent years (e.g. 2019/2020) when episodic large stratospheric ozone depletion has followed anomalously cold stratospheric winters [10]. However, unlike in the Antarctic, these events occur during early spring when most organisms are still protected by sea ice or snow cover.

Changes in the stratosphere driven by ozone depletion have also been clearly shown to cause seasonally dependent shifts in near-surface patterns of wind, temperature and precipitation [39–42]. Knock-on effects on warming of oceans and melting sea ice cover have been investigated [40–49], but many uncertainties persist [50] as the effects of ozone depletion on weather patterns are occurring against a backdrop of climate change. Collectively, these changes have led to increased variability of weather and climate, which is most pronounced in the polar regions [51]. As documented in our previous assessments and elsewhere, these shifts in weather and climate have had pronounced impacts on many Antarctic organisms, from tiny moss and cushion plants to wandering albatross [12, 34, 52, 53].

Since our last Quadrennial Assessment, extremes have occurred in both ozone depletion and climatic events that have led to observed or potential effects on plants and animals in polar regions (Table 2). Specific findings include:

- During spring 2019, the Antarctic stratosphere was strongly disturbed by meteorological influences from upward-propagating atmospheric waves [54–56] resulting in a small ozone hole*.* These stratospheric conditions played a role in enhancing prolonged drought over the 2019/2020 austral summer that exacerbated the unprecedented wildfires in eastern Australia [57–64]*.* Effects on stratospheric chemistry following the wildfires led to wider changes in both the chemical composition and temperature of the stratosphere across southern mid-latitudes [18, 65–73]*.* Strong vertical and horizontal gradients in the ozone concentration of the Antarctic upper troposphere during the austral spring potentially delayed the subsequent effects on surface climate [32].The role of ozone depletion in modulating the dynamical coupling between the polar stratosphere and the surface at lower latitudes for this particular season is still under investigation. Nevertheless, it appears likely that the combined effects of climate change and ozone depletion could have impacted both the timing and magnitude of these wildfires with considerable consequences for ecosystems in this region.
- In contrast to 2019, a strong and persistent Antarctic ozone hole occurred in 2020 and 2021 [54, 74–76] and this led to record surface UV irradiances at several sites across East Antarctica during early summer. It has been suggested that the Australian wildfires that occurred during the previous summer contributed to this strong ozone loss [18, 54–56, 71, 72, 74]. There is evidence that increased ozone depletion has tended to delay the annual breakdown of the Antarctic stratospheric vortex [77]. Modelling suggests that increasing greenhouse gas concentrations also favour a more persistent vortex [78], as well as reducing the likelihood of a weaker vortex [58]. While concentrations of ozone-depleting substances (ODSs) remain elevated, later seasonal persistence of the Antarctic vortex could expose organisms to higher UV irradiances at times of year when young animals are born/hatch and when plants are actively growing. The loss of protective snow cover could exacerbate these effects [34].
- Since our last assessment there have been two widespread heatwave events in Antarctica, the first in summer 2019/2020 when heat records were broken around the continent [63]. In March 2022 (autumn) extreme temperatures, almost 40 ˚C higher than normal, were reported as an atmospheric river, or plume of warm, moist air, moved onto the Antarctic plateau. Heatwaves such as these accelerate melting of icebanks [79], potentially exposing vegetation to high springtime UV-B radiation from which they have previously been protected [36]. The impacts of these heatwaves and the subsequent ice melt have been poorly studied in Antarctica in part due to the lack of environmental monitoring with networks of sensors tracking temperature and climate variables at appropriate scales. This lack of data is well illustrated by the recently published global maps of soil temperature [80], which exclude Antarctica. Warming temperatures on the Antarctic Peninsula are opening up ice free areas [79] causing the expansion of vascular plants [81] and increasing the possibility of new plant and animal species invading the continent [21, 82]. As in the Arctic, there are examples of both plant expansion (i.e. “greening”, [81, 83]) and death of plants by drought (i.e. “browning”, [84–88]). Heatwaves may also be particularly detrimental to mosses, as they survive by creating warm microclimates in Antarctica’s cold environments but this may become a disadvantage as air temperatures increase [89].
- In the Arctic, unprecedented low total column ozone values occurred in the 2020 boreal spring [90, 91] due to strong stratospheric ozone depletion, and this resulted in record-breaking high solar UV-B irradiances [92, 93]. These conditions were promoted by weak tropospheric wave activity [90, 94], associated with anomalous sea surface temperature in the North Pacific [33], which caused the stratospheric vortex to become large and stable*.* Heatwave conditions that occurred in the Siberian Arctic in early 2020 [95] appear to have been aided by atmospheric circulation patterns that were affected by the strong ozone depletion [33, 94]. Ozone depletion in March 2020 may also have aided the prevailing reduction of sea ice in the Arctic Ocean bordering Siberia [96]. As indicated above, most Arctic organisms are currently protected by snow and sea ice at the time of maximum ozone depletion and high UV radiation conditions at this time of year (i.e. early March to mid-April 2020), but changes in snow and ice cover resulting from climate change could increase exposure to UV radiation.

**Table 2:** Summary of environmental effects of stratospheric ozone changes and concurrent climate extremes from 2018 to 2022.

Ozone effects	Climate extremes and associated effects	Plant responses	Animal responses, including humans
**Antarctica and Southern Hemisphere (September 2019–February 2020) **			
Anomalously small ozone hole	Wildfires in Australia produced aerosols that caused ozone depletion and black carbon particles that accelerated snow melt [76]	Widespread loss of plant biomass in Australia [97]	Loss of human life and adverse health effects; loss of domestic animals and wildlife [98–101]
	Heatwaves in Antarctica [102]	Additional snow melt that caused temporary greening of some previously moribund moss beds in East Antarctica [53, 103] Vascular plants on the Antarctic Peninsula appear to be faring better than mosses under global warming and the grass *Deschampsia antarctica* appears to be quite tolerant of *in-vitro* high temperature shock treatments [104] Extreme summer marine heatwaves increased chlorophyll *a* (an indicator of the abundance of phytoplankton) in the Southern Ocean [105] Hotter and longer heatwaves increased the mortality and decreased post-heatwave growth rates in the Southern Ocean diatom *Actinocyclus actinochilus* relative to milder, shorter heatwaves [106]	Functional thermal limits for the Antarctic sea urchin (*Sterechinus neumayeri*) were determined under simulated marine heatwaves. Key biological functions vary in their thermal sensitivity and in their responses to different rates of warming [107]
**Arctic (January – April 2020)**			
Anomalously large ozone depletion [8]	Heatwave in the Siberian Arctic; accelerated loss of sea ice [96]	Permafrost warming and thaw lead to landscape changes (retrogressive thaw slumps) and increased greenhouse gas emissions [108]	Negative impacts on organisms that depend on sea ice; positive impacts on animals that thrive in open oceans [95, 109, 110]
**Antarctica (November – December 2020 and November – December 2021)**			
Persistent ozone hole producing anomalous surface UV irradiance [76]. See also [10]		High potential for excessive exposure to UV radiation as plants emerge from under winter snow [82]. Earlier snow melt, meaning more exposure, which coincided with extreme maximum UV Index	High potential for excessive exposure to UV radiation as animals return to breeding sites in spring and early summer [111]. Reductions in Antarctic sea ice [21] will result in higher exposure to UV radiation in the water column

The factors that affect the size of the Antarctic and Arctic ozone holes each spring bring widespread climate impacts that can extend far beyond the polar regions. For example, Antarctic ozone depletion varies with the phase of the southern annular mode (SAM). The SAM phase has been linked to the black summer (2019/2020) bushfires in Australia, which produced aerosols that contributed to further ozone depletion and smoke particles, which accelerated snow melt in New Zealand and Antarctica. Note that the assessment of the environmental impacts of heatwaves and anomalous ozone dynamics has been extremely limited due to the COVID-19 pandemic that prevented many planned scientific visits to remote polar regions

### Interactive effects of extreme climate events and UV radiation extending beyond polar ecosystems

**Fig. 1:**
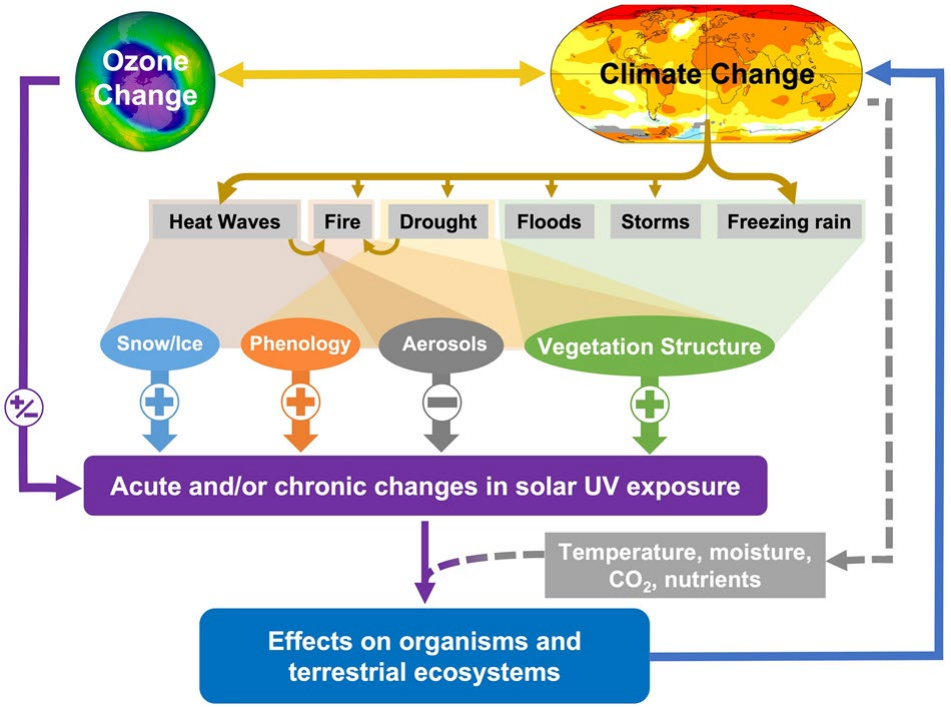
Pathways by which extreme climate events (ECEs) driven by changes in stratospheric ozone and climate can affect exposure of terrestrial organisms and ecosystems to UV radiation. Changes in stratospheric ozone and climate interact to influence the frequency and intensity of a number of ECEs (upper-most grey rectangles). These ECEs in turn affect atmospheric and surface intermediaries (multi-coloured ovals connected with ECEs by overlapping shaded regions), which can increase ( +) or decrease (-) the solar UV radiation reaching terrestrial organisms and ecosystems. Solid arrows show direct mediation by climate, ozone and UV radiation on ECEs and potential interactive and feedback effects. Dashed arrows show chronic effects of climate change factors

Globally, extreme climate events (ECEs^1^) are increasing in frequency and severity with climate change and are projected to become even more prevalent in the future as the climate continues to change [20]. Examples of ECEs include stronger storms and tropical cyclones, catastrophic floods, protracted droughts, anomalous heat waves and freezes, and more intense wildfires [113–118]. ECEs cause long-term disruption to ecosystem structure and function [119–122] and occur against a backdrop of more gradual changes in the environment (e.g. rising surface temperatures and atmospheric carbon dioxide (CO_2_) concentrations). These disruptions to ecosystem function can exacerbate the deleterious effects of ECEs on plants and animals [123]. Extreme climate events also alter the amount of UV radiation reaching terrestrial ecosystems (Fig. 1). These changes in UV radiation can occur over short or long timeframes, which can then lead to acute or chronic effects on ecosystems, respectively. The changes in solar UV radiation together with other environmental factors (e.g. temperature, availability of moisture) may affect biodiversity, productivity, emissions of greenhouse gases [124–126], and ecosystem carbon storage [123]. For example, fires, floods, and tropical cyclones (hurricanes) all create openings in forest canopies [127, 128], driving subsequent adjustment in the understorey vegetation to an acute or chronic increase in incident solar radiation; these increases in solar radiation are often accompanied by increases in temperature and decreases in soil moisture [129–131]. There is also an associated increase in the amplitude of fluctuations in these abiotic factors. Some plant species (e.g. shade-adapted specialists) may not be able to adjust to this new environment and will go locally extinct. However, other plant species can respond quickly to these environmental changes [132–134] and may increase in abundance. With respect to UV radiation, some plant species can respond rapidly to increases in amounts and variability in solar UV radiation through the production and accumulation of UV-protective pigments [131, 135] (Sect. 3), and these attributes may allow these species to be successful in the changing conditions. From an ecosystem perspective, fires and hurricanes are among the most disruptive examples of ECEs as they can cause the loss of productivity and biodiversity, and increase the emissions of GHGs [136–138], which can be enhanced by UV radiation (Sect. 6).

The disruptive nature of ECEs also opens up the remaining ecological communities to invasive species, which can further destabilise these systems [12]. For example, certain invasive species that can tolerate high solar radiation and colonise open habitats may displace some native, specialised or endemic species [139]. To what extent differences among plant species in their tolerances to UV radiation influences species invasions into high UV environments remains unclear [140, 141]. Recovery of ecosystems from these ECEs will largely depend on the species that colonise the more open habitats created, and their biodiversity value and traits that support ecosystem function [142].

Wildfires and droughts affect the amount of UV and photosynthetically active radiation (PAR; 400–700 nm) reaching terrestrial ecosystems due to increasing aerosols from smoke and dust, and volatile organic compounds released by plants [143]. These atmospheric changes not only reduce PAR and UV radiation, but also change the spectral composition of sunlight at ground level [144]. Importantly, changes in air quality resulting from fires and droughts can occur well beyond the location of these events [145–147]. Thus, these conditions arising from fires and droughts can potentially affect photosynthesis and light-driven development in plants [148], as well as litter decomposition and GHG emissions in ecosystems [19] not directly impacted by these extreme events (Sect. 6.1).

## Effects of UV radiation and climate interactions on plants and animals

While moderate UV-B irradiance serves as an informational cue that facilitates the normal regulation of plant growth and metabolism, exposure to excessive UV radiation, and in particular short-wavelength UV-B radiation, can have deleterious effects on terrestrial organisms [e.g. 150]. As sessile, photosynthetic organisms, plants require sunlight for their growth and reproduction, but this also means that they can receive a large cumulative amount of solar UV radiation over their lifetime. This cumulative amount would have been very high in the extreme UV irradiation conditions that would have occurred without the Montreal Protocol [1]; however, because of its implementation appreciable reductions in photosynthesis in terrestrial plants have been avoided. High UV irradiance conditions would also likely impair growth with severe consequences for global carbon storage and climate [3, 10] (Box 1). Under current climate conditions, and in most regions of the world, land plants appear to show adequate protection against UV radiation that limits the deleterious effects of moderate UV-B radiation. There are physiological similarities in responses to adverse conditions (stress), that may determine the extent to which plants can tolerate increased UV radiation in combination with other abiotic factors (e.g. temperature, drought, elevated CO_2_) that occur simultaneously. In this section, we highlight recent progress in identifying the mechanisms by which plants perceive and respond to UV radiation. These findings allow us to better assess the impacts of changes in UV radiation, plant response to rapid increases in UV radiation (as occur following many ECEs), and how UV radiation interacts with environmental stresses (e.g. climate change) to modulate their growth and productivity.

In contrast to the abundant literature on the effects of UV-B radiation on terrestrial plants, far less attention has been paid to the effects of UV-B radiation on terrestrial animals. What research there is on animals, typically addresses vision in the UV-A waveband and its effect on behaviour [150], and the application of these findings for controlling insect pests and pollinators of certain crops [151]. One exception is the increasing research, largely focussed on agricultural systems, showing that terrestrial invertebrates, including mites [152, 153] and insects such as aphids [154–156], are vulnerable to direct damage from UV-B radiation. There are interesting parallels between invertebrate and plant responses to UV radiation; for example, in the role of DNA-repair [157, 158], antioxidant metabolism [159–162] and pigments [155, 156, 161] in conferring UV-protection. This includes evidence that mites can obtain UV-protective compounds by consuming pollen [160]. It is also clear that avoidance behaviour plays a major part in reducing the exposure of invertebrates to solar UV radiation [152–154, 163].

### Perception and response of plants to changing UV radiation

The need to better understand how organisms respond to elevated UV-B radiation, as occurs with stratospheric ozone depletion, stimulated research that eventually led to the discovery of a UV-B photoreceptor in plants (UVR8, which stands for Ultraviolet Resistance Locus 8) [164]. It is now well-documented that UVR8 mediates a number of plant responses to changes in UV-B radiation in the environment. Recently, UVR8 has been found to operate over a spectral region extending through the UV-B and part of the UV-A radiation wavebands [165]. Thus, variation in solar UV radiation attenuated by the stratospheric ozone layer (which screens UV radiation up to *ca.* 335 nm) is well matched to the action spectrum of UVR8 [166, 167]. This might suggest that the evolution of UVR8 allowed plants to perceive and respond to environmental cues related to changes in stratospheric ozone.

The UVR8-signalling pathway likely evolved very early in the transition of plants from aquatic to terrestrial environments [168–170]. Two overlapping signalling pathways for UV responses (UVR8/WRKY36/HY5 and UVR8/COP1/SPA-HY5 pathways) have been conserved during the evolution of green plants [170, 171]. These pathways regulate a series of genetic transcription factors that affect accumulation of flavonoids, functioning of the plant hormone auxin, and growth (i.e. through inhibition of elongation of lateral roots and hypocotyls [172]). Subsequently, diversification of signal transduction to increase crosstalk with other signalling pathways that control the production of additional secondary metabolites, such as brassinosteroids (hormones involved in plant development), enabled fine-tuning of tolerance to UV radiation in photosynthetic organisms. Specific responses in plants that are involved in their acclimation to UV radiation include: the accumulation of flavonoid pigments as UV sunscreens, shorter stature with increased branching, and smaller leaves with thickened cell walls. These changes together with a more conservative strategy (i.e. slower but more efficient growth, photosynthesis, and water loss [173, 174]) collectively mitigate the potentially deleterious effects of current levels of solar UV radiation on plants.

Among the diverse functions of phenolic compounds in growth, development and reproduction, certain flavonoids and related phenolic acids (e.g. hydroxycinnamic acid derivatives) screen UV radiation in plant tissues and are therefore central to plant UV-acclimation responses. The accumulation of these compounds in leaves, flower petals and pollen is temperature dependent but is also driven by UV-B radiation [135, 175, 176]. Flavonoids fulfil many additional roles in plants, in that they are involved in ameliorating biotic and abiotic environmental-stress, regulating the transport of certain hormones (i.e. auxin) and are required in many species for successful germination and growth of the pollen tube on the stigma of flowers, where they participate in cell signalling and recognition [177–180]. There is also evidence that greater accumulation of flavonoids in pollen grains improves their germination (e.g. in *Clarkia unguiculate*; [181]) and flavonoids function in UV screening in pollen, which is essential to maintain viability [182, 183]. Additionally, flavonoid glycosides (quercetins and kaempferols), hydroxycinnamic acids and anthocyanins in leaves and pollen act as strong antioxidants, and, as such, they scavenge reactive oxygen species (ROS) produced by abiotic stressors such as excessive solar radiation, including UV-B radiation [178, 184].

In assessing plant acclimation to increased UV radiation, it is relevant to consider responses to short-term, rapid fluctuations in UV radiation—as would occur with changing cloud cover or from day-to-day during the break-up of the stratospheric ozone hole—as well as to the longer-term (i.e. decade-scale changes that occur from anthropogenic changes in stratospheric ozone together with climate). The patterns of these responses can be used to evaluate whether plants’ epidermal UV screening and photoprotection principally acclimate to immediate changes in UV radiation or if plants mainly rely on other mechanisms that allow trans-generational improvements in protection against UV radiation (i.e. genetic adaptation or epigenetics). There is increasing evidence that the accumulation of photoprotective compounds (including flavonoids, hydroxycinnamic acids and carotenoids) tracks seasonal and even daily variation in UV radiation [185–190]. In general, the magnitude of diurnal changes in UV screening is less than those that occur during the development of leaves. Diurnal changes in UV screening can, however, be of comparable size to the variation in screening that results from day-to-day fluctuations in UV radiation and temperature [176, 186, 191]. Rapid acclimation of UV screening to short-term changes in UV radiation indicates a high level of phenotypic plasticity and suggests that many plants can acclimate to short-term fluctuations in UV irradiance arising from transient reductions in stratospheric ozone, reduced cloud cover or certain ECEs (Fig. 1; Sect. 2.2). In fact, a comparison of 629 taxa growing together at high-elevation and high-latitude locations subject to strongly contrasting UV irradiances, found phenotypic plasticity in epidermal UV screening according to their immediate growing microenvironment, and this outweighed any differences in adaptation arising from their evolutionary history under disparate climates [192]. Similarly, the importance of the local environment over the place of origin is also highlighted by experiments where species and populations are grown in the same location and habitat (i.e. common-garden experiments [193]).

Although the capacity for rapid acclimation may be advantageous for adjusting to short-term environmental variability, high phenotypic plasticity may interfere with the capacity for genetic adaptation to changing conditions over long time periods [194–196]. Understanding the relative importance of phenotypic plasticity *vs*. genetic adaptation is needed to evaluate the consequences of climate change-induced range shifts that expose plant species to UV irradiances that might be beyond those experienced in their historic ranges (Sect. 4.1).
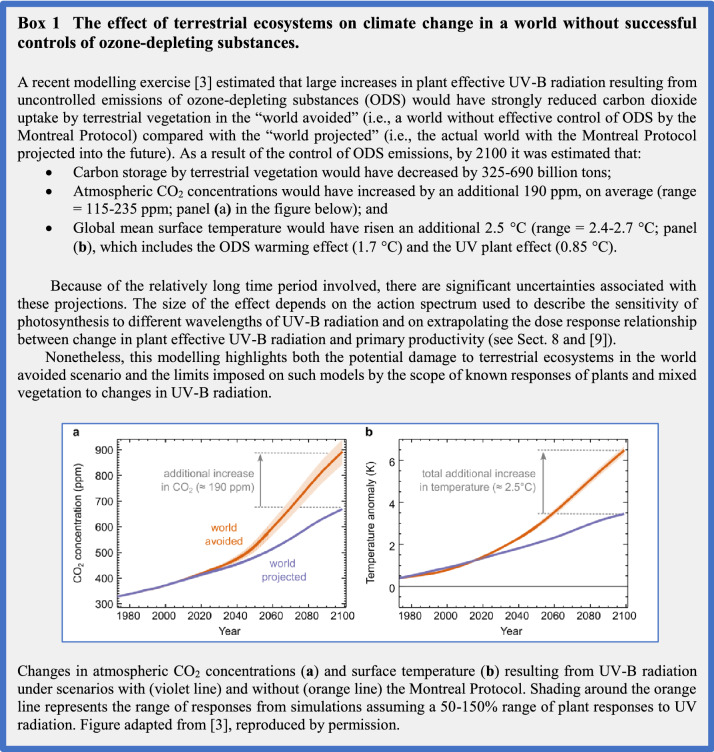


Studies examining the mechanisms by which native plant species tolerate naturally high UV-B environments can provide insights into the range of adaptive responses exhibited by plants to UV-B radiation. For example, *Rheum nobile* (Sikkim Rhubarb), an herbaceous plant that grows above 4000 m on the Tibetan Plateau, has large translucent flower bracts containing high concentrations of flavonoids, which form a protective cover over its flowers. This species can, thus, attenuate UV-B radiation within its floral tissues to similar levels across its elevation range [197]. In the same region, other herbaceous plants, such as *Megacarpaea delavayi* (a wild mustard [198]) and five species in the genus *Saussurea* (thistle-like plants in the sunflower family [199]) have a rapid DNA-repair mechanism to mitigate the damaging effects of high UV-B irradiances. Comparative genomic analysis of 377 Tibetan peach populations showed that the expansion of SINE retrotransposons (genetic variations that regulate gene expression), promotes adaptation to UV-B radiation [200]. These, and other evolutionary adaptations of specialist alpine species to extreme UV radiation conditions indicate how plants in general might adapt to high UV-B irradiances. However, the rate of changes in UV irradiance as a result of ozone depletion or climate change is likely to outpace the rates of adaptation in many species, especially long-lived perennials such as trees. Additionally, plants endemic to high elevations often have limited distribution ranges and abundances, and may be among the most vulnerable to habitat loss due to climate change.

### Proxies for past solar UV irradiance based on acclimation responses of modern-day plants to UV radiation

Because many of the phenolic UV sunscreens accumulated by plants are resistant to decay, it may be possible to infer historical changes in solar UV radiation from tissue samples of plants that have been preserved in herbaria or in sediment cores. Herbarium specimens offer the potential to retrospectively infer past environmental conditions by assessing how plant traits have changed over the period of their collection (usually decades). However, to be reliable proxies for UV radiation, herbarium specimens must be sampled in a consistent and unbiased manner over time (See [201] for a full discussion of necessary procedures). If these protocols are followed, and if other factors that can modify flavonoid and anthocyanin accumulation in plants (e.g. shading, changes in temperature, availability of moisture or total solar irradiance) are accounted for, one could associate trends in pigmentation of thermostable compounds with historical changes in UV radiation.

Over longer time frames the effects of major global events such as changes in solar activity, volcanic eruptions, or reversals in the Earth’s magnetic field (e.g. at the Laschamps Excursion 42,000 years ago) might be examined through changes in the phenolic composition of pollen or spores. For example, the hydroxycinnamic acid *para*-coumaric acid, which is preserved in fossilised sporopollenin (a compound that forms the outer wall of spores and pollen), continues to be the focus of research seeking such a proxy for UV-B radiation over geological time periods. However, before it can be reliably used it is necessary to ascertain the action spectrum of its response to UV radiation, its rate of degradation, the response time of its synthesis, as well as the consistency of response among species and over time [202]. In the case of fossilised pollen from *Nitraria* (a steppe plant) and conifers, chemical signatures have been shown to differ from those of contemporary (extant) pollen in a predictable and consistent manner such that stable relationships can be modelled [203].

### Interactive effects of UV radiation and climate change factors

Ongoing changes in climate, together with associated changes in plant species distribution, are exposing wild plants, forests and crops to new combinations of UV radiation and other climatic conditions [10, 124]. Combinations of particular concern are high UV-B irradiance and drought or temperature, as climate change is increasing the frequency and severity of heat waves and droughts, and these events frequently coincide with high UV radiation, particularly at mid to low latitudes [204].

There are marked similarities in the acclimation responses of plants to increases in UV radiation and drought. A recent meta-analysis found these two sets of responses to be generally consistent irrespective of whether experiments were performed in controlled environments (i.e. growth chambers or greenhouses) or in the field [205]. In general, when plants are exposed to co-occurring drought and increased UV irradiance, the accumulation of defence compounds (e.g. proline and secondary antioxidants, such as flavonoids and anthocyanins) and other stress responses (e.g. decreased leaf area, reduced stomatal opening) is enhanced. Thus, the combined detrimental effects of these stressors on plant function are milder (i.e. reduced production of stress‐associated malondialdehyde (MDA) and reactive oxygen species (ROS)), and this reduces the negative effects on photosynthesis and biomass production [205, 206]. The response of plants to increased UV radiation may therefore confer cross-protection against drought [205, 207] and mitigate some of the detrimental effects of drought on plant growth and productivity, unless both stress factors are excessive. Further, it has been postulated that plants may use UV radiation as a signal of impending drought [208]. The functional association between exposure to drought and UV radiation exposure appears to involve common physiological defence and acclimation responses [208, 209]. For example, multiple studies have shown that overexpression of protective pigments in plants results in enhanced protection against both drought and UV-B radiation [210, 211]. UV-B radiation can even be exploited for seed priming, resulting in enhanced expression of drought tolerance of plants grown from such UV pre-treated seeds [212]. Certain agricultural practices may also negatively impact crop tolerance of both UV radiation and drought. For example, growth allocation to roots relative to shoots often increases in drought-stressed plants, as well as those exposed to high solar UV-B radiation (i.e. increased root:shoot ratios), but high nitrogen availability has the opposite effect on root–shoot allocation [213].

High UV-B irradiance often co-occurs with high temperatures. A recent study of a commercial tomato cultivar (*Solanum lycopersicum* cv. Money Maker) compared plants transferred under near-ambient solar UV radiation to those placed in a UV exclusion treatment in the field. Exposure to UV-B radiation led to partial closure of leaf stomatal pores, reducing transpiration and evaporative cooling, and thus increasing leaf temperature by up to 1.5 °C [214]. These findings are relevant in warmer climates where even small increases in temperature may have substantial consequences for survival of crops [215], as high temperatures are well-known to negatively affect photosynthesis and growth of many plant species. More broadly, a recent meta-analysis across terrestrial, freshwater and marine plants, algae and animals [216] showed that any negative effects of UV-B radiation can be somewhat compensated for by elevated temperatures, although this depends on the habitat and organism involved. This positive effect of warming appears to be restricted to cool climates where organisms often function at temperatures below their physiological optima, and thus is not expected to occur in environments approaching the thermal and physiological limits of organisms [216]. Given the current context of global warming, more detailed temperature and UV-radiation dose–response studies are required to fill this knowledge gap. Furthermore, the scope of such studies needs to go beyond crop yield, as early evidence shows that interactive effects of heat and UV radiation can also affect crop quality [217] (Sect. 5.2).

Apart from high temperatures, the effects of UV radiation on plants can also be modified by low temperatures, and climate change is expected to increase the incidence of extreme cold events in some regions [20](Sect. 2.2). In studies with the model plant *Arabidopsis thaliana*, the synthesis of flavonoids is strongly enhanced in response to low temperatures (4/2 °C, day/night) compared to moderate temperatures (18/20 °C), just as it is by UV radiation. Where plants are simultaneously exposed to both cold and UV radiation, complex interactive effects are observed, with UV-B decoupling flavonoid accumulation from gene expression, indicating post-translational regulation [218]. Low temperatures and UV-B radiation also produce a shift in the composition of flavonoid glycosides from kaempferols to quercetins [176]. The shift in composition towards quercetin synthesis at low temperatures suggests an enhancement in antioxidant function [176, 219], which could increase overall plant hardiness.

Temperature is a cue for many organisms, controlling their seasonal development (i.e. phenology). Changes in thermal regime, such as periods of extreme heat or cold or even an absence of cold temperatures, can disrupt the timing of growth, reproduction, and other aspects of phenology [220, 221]. Temporal shifts in phenology can also change the seasonal timing of exposure to UV radiation, as solar UV radiation varies at high-to-mid-latitudes over the course of the year. Shifts in phenology due to changes in climate and UV radiation may result in new combinations of biotic interactions (i.e. competitors and pests; Sect. 4.1) and abiotic stresses that may be outside the tolerances for some species. For plants, these new combinations of abiotic stresses can have detrimental effects on their growth and survival even though each individual stressor may have a negligible effect [222].

Complex effects on plants may also occur when other environmental factors interact with UV radiation. Recent studies have revisited the interactive effects of UV radiation and increased nitrogen deposition [223], ozone pollution [15, 224] and elevated atmospheric CO_2_ concentration, where short-term stimulation can be outweighed by long-term downregulation of photosynthesis [225], as noted in our previous assessments [12, 226]. Elucidation of the interactive effects of UV radiation and these other environmental factors is necessary to improve our ability to model and assess the effects of UV radiation on the carbon sequestration of terrestrial vegetation in a changing climate (e.g. Box 1).

## Species distributions and biodiversity

Maintaining the wide variety of plants, animals, and microorganisms in terrestrial environments (i.e. biodiversity) is essential for ecosystem health, stability, and valuable services provided to humans. The loss of biodiversity can occur directly (e.g. hunting or harvesting) or indirectly (e.g. loss of habitat, climate change, and invasive species). While considerable attention has been given to the effects of climate change on biodiversity [21, 227, 228], far less is known about how solar UV radiation might interact with climate change to influence species distributions and diversity in ecological communities. We examine these effects from available studies and evaluate how the UV radiation exposures of species can potentially change as their distributions shift in response to climate change.

### Potential effects of climate change and UV radiation on shifting species distributions

Plant and animal species are migrating or shifting their distribution ranges to higher elevations and latitudes in response to ongoing changes in climate [229–231]. As species occupy higher elevations and latitudes, they may encounter increased or decreased UV radiation, respectively, because of the natural gradients in solar UV radiation that occur with elevation and latitude (Table 1). Some plants and animals are also shifting their ranges in the opposite direction, viz., towards lower elevation (lower UV radiation) and latitude (higher UV radiation), to avoid the increased seasonality of temperature at higher latitudes [232, 233]. How species respond to novel combinations of UV radiation and multiple climatic conditions has direct implications for how they will interact with other species, including their pests and pathogens (Sect. 5.3), with consequences for biodiversity [e.g. 235].

#### Latitudinal change

**Fig. 2:**
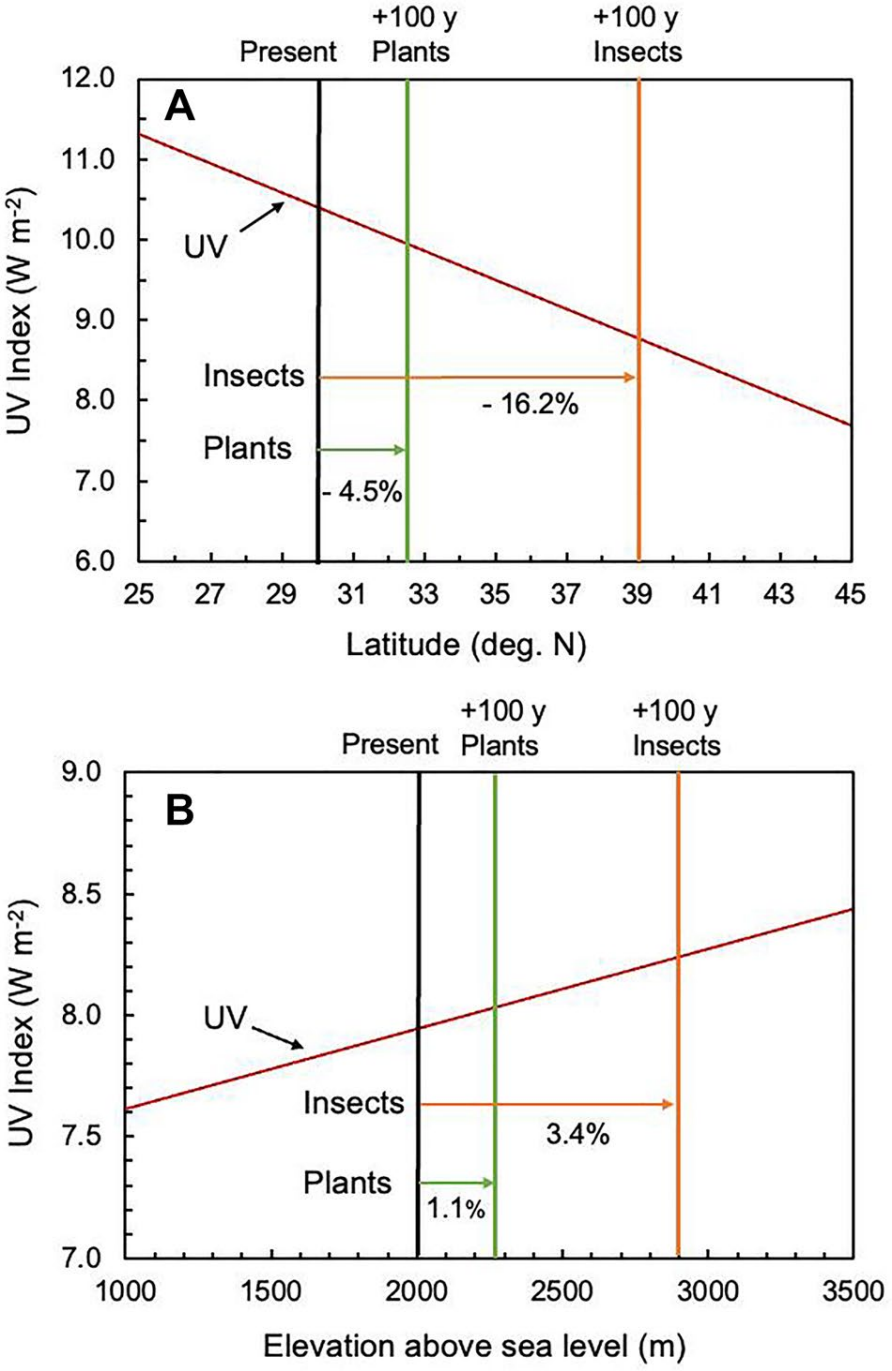
Potential changes in exposure to UV radiation as plants and insects migrate to higher latitudes and elevations with climate change. Panel **A** shows the estimated changes in UV radiation as plants and their herbivorous insects migrate poleward after 100 years (y) of climate change. UV radiation data are simulated midday summer (June 21) UV irradiances (here reported as UV Index; red line) based on stratospheric ozone levels in 1980 at sea level (radiative transfer model TUV; [263]). Horizontal arrows show distances migrated for herbaceous plants (green arrow) and plant-eating insects (orange arrow) originating from 30° N after 100 years of climate change assuming maximum rates of migration and average climate velocity for 2050–2090 (from [235]). Panel **B** shows the simulated midday summer (June 21) clear sky UV Index changes with elevation in the European Alps (46° N latitude; red line) and the estimated changes in UV irradiance for plants (green line) and insects (orange line) as they migrate from 2000 m to higher elevations after 100 years of climate change, assuming average current rates of leading edge migration for Western European montane plants (28.2 m/decade) and insects (90.5 m/decade) [264]

While the changes in UV radiation received by plants and animals resulting from latitudinal shifts in ranges are generally rather modest, they may affect terrestrial ecosystems and biodiversity. For instance, if one assumes species migrate at their maximal rates to keep pace with climate change (i.e. their average climate velocity for the period 2050–2090; [235]), the UV irradiance under clear sky conditions for herbaceous plants would decline by 4.5%, while that for more mobile plant-eating insects would decline by 16.2% after a century of climate change (Fig. 2A).

Plants encountering reduced UV-B irradiance resulting from range shifts would likely reduce their levels of UV-protective compounds (i.e. epidermal flavonoids and other phenolic compounds) [236, 237]. The multiplicity of roles performed by these plant secondary compounds could, in turn, make some plants more vulnerable to herbivores [238, 239] (Sect. 5.3) as some of these chemicals serve as deterrents for insect herbivores.

Accelerated loss of biodiversity will likely occur as climate change continues to exert its effects on range shifts on plants and animals. Plants use both temperature and day length (photoperiod) as environmental cues for regulation of phenology (flowering, dormancy, budburst, etc.). Trees with long generation times may be especially vulnerable to extinction because they have limited opportunities to genetically adapt to a changing photoperiod and their environmental cues such as UV radiation may be mismatched with their new environment [240–242]. At present, it is unclear how changes in UV radiation in combination with climate change will affect species migrations and adaptation as experimental and modelling data are not yet available to quantify and fully assess the risk of these interactive effects.

#### Elevational change

For many montane ecosystems, climate change is resulting in the migration of lower elevation species to higher elevations. Climate change is also reducing the envelope of suitable habitats for high elevation alpine species to survive, while increasing competition against emigrating species from lower elevations [21, 243]. However, like latitudinal shifts towards the equator, elevational distribution changes also occur downslope for some species [232, 233]. For species shifting their ranges to higher elevations, their exposure to solar UV radiation would be expected to increase, assuming no change in cloud cover (Fig. 2B). Further, reduced snow cover due to warmer temperatures exposes organisms to fluctuations in temperature and solar radiation, including UV-B radiation [244].

High elevation alpine plants often have heightened accumulation of UV-screening compounds and herbivore defence [140, 245–248]. Across a diversity of plant species from alpine and subalpine zones in Bulgaria, improved photoprotection has been found to effectively prevent greater DNA damage caused by increased UV radiation at higher altitudes. These mechanisms were sufficiently effective since plants growing at the highest elevations had fewer UV-induced DNA dimers than those at lower elevations, with grasses (Family Poaceae) least susceptible to UV-induced DNA damage among a wide diversity of plant families tested [249]. Nevertheless, as species migrate to high elevations, more resources may be allocated towards protection against UV radiation, and this could in turn divert resources away from growth, which could then reduce competitive ability [250, 251]. Depending on the availability of suitable habitats at higher elevations, these changes in species interactions have the potential to negatively affect biodiversity [252] by shifting the balance of competition between species [253, 254].

While climate change is causing many species to migrate to higher elevations, these climate change-induced shifts in distribution ranges are often most pronounced for non-native, invasive species [255–257]. At present, it is unclear if UV radiation affects native and non-native invasive species differently [140, 141, 258–260]. However, invasive species are generally considered to exhibit greater phenotypic plasticity to new environments than native species, although this may depend on availability of resources [261, 262]. In some cases, invasive species have been found to alter their production of UV screening compounds to a greater degree than native species [140]. This flexibility may allow non-native, invasive species to occupy new habitats more rapidly than native species and, in some cases, outcompete endemic alpine species [234].

### Assessing the risks to biodiversity from the interactive effects of UV radiation and climate change

Climate change can cause declines in biodiversity by reducing the availability of suitable habitats for species and by differentially shifting their distribution ranges, which then disrupts species interactions and ecosystem function. If species cannot keep pace with climate change, then populations will decline leading to a loss of biodiversity. In this context, species distribution models (SDMs) are used to determine how climate change will affect future habitat suitability of species through changes in key abiotic drivers. These models can be used to inform species conservation as well as management for plant production in agriculture and forestry [265].

Several studies have shown that the inclusion of solar UV-B radiation in models forecasting future distribution ranges of ecologically and agriculturally important crop and tree species improves their statistical predictive power [266–272]. These models are based on different scenarios of climate change (i.e. IPCC scenarios of greenhouse gas emissions) and create projections based on correlative relationships between climate and species occurrence. These projections suggest that the ranges of some species of native plants from open, dry habitats found in arid and semi-arid shrub-steppe biomes will expand to higher elevations [267–272], while the ranges of willows and other related species from wetter habitats will contract [266].

Some studies of plants native to China and central Asia include UV-B radiation among the potential explanatory climatic variables that contribute to species distributions, sourcing data from the global climatology [273]. Future habitat suitability estimated using Maximum Entropy models (MaxEnt; models that apply basic machine learning algorithms to resolve environmental conditions where the species is present across its distribution) reveal incident UV-B radiation together with precipitation and temperature as significant correlates of species occurrence. While these models do not identify the mechanisms underlying these results, the findings suggest that such models could be useful in assessing risks to biodiversity, as well as providing information on potential species distributions and suitable habitats for conservation and planting crops for different scenarios of climate and solar UV-B radiation.

Despite the inclusion of UV-B radiation among significant climatic variables in some studies of species distributions [266–272], most modelling studies to date do not include UV-B radiation and its interaction with other abiotic stressors as potential constraints on species distribution. As more detailed UV-B databases become available, it is likely that UV-B radiation will more routinely be included among climatic variables used to predict species occurrence and changes in biodiversity. As well as species distribution, climatological data that include regional UV-B irradiances can be applied to study whether climatic trends correlate with patterns in plant functional traits among species. For example, a large-scale study of 1192 grassland species found that UV radiation was negatively correlated with leaf size across the Mongolian and Tibetan Plateau [274], while leaf shape, reflectance, and thickness have also been found to covary with UV-B radiation along environmental gradients [275–277].

## Effects on agriculture and food production

Some of the earliest concerns raised over stratospheric ozone depletion and the accompanying increase in solar UV-B radiation considered the potential for reductions in crop productivity and compromised food security [278, 279]. A prior assessment [38] using results from field studies conducted at high latitude locations indicated that plant productivity declines by about 3% for every 10% increase in plant effective UV-B radiation (i.e. UV-B radiation weighted according to a generalised plant action spectrum [280]). These findings implied that the projected increases in solar UV radiation with changes in stratospheric ozone and climate, assuming full compliance with the Montreal Protocol, would have minimal effects on agricultural productivity. However, few experimental studies to date have been conducted on species growing in those regions with the highest natural levels of UV-B radiation on Earth (i.e. the tropics and high elevations). Previous studies also tended to focus on crop productivity but paid less attention to the effects of UV radiation on food quality. The effects of UV radiation on agroecosystems also extends beyond the direct effects on crop plants, as UV radiation can influence pest–pathogen interactions and the persistence and effectiveness of biocides and agricultural pollutants. The management of solar radiation in greenhouses and advances in artificial UV lighting are exploiting some of the beneficial effects of modest exposures to UV radiation to improve food quality, enhance plant defences against pest and pathogens, and contributing to more sustainable agricultural practices [281].

### Agroecosystems vulnerable to changes in UV radiation and climate

As noted in our 2019 Update Assessment [282], and in other reports [283, 284], most field research to date on the effects of UV radiation on crops has been concentrated on regions outside the tropics and at lower elevations. The tropics extend over approximately 33% of the Earth’s land surface [285] and harbour a vast reservoir of biodiversity [286] that provides critical resources and essential services for agriculture and food security [287]. Thus, tropical agroecosystems warrant further attention to safeguard a sustainable future for life on Earth.

Because the projected recovery of stratospheric ozone is highly dependent on changes in GHG concentrations and lifetimes of ODS, there remains some uncertainty about how UV-B radiation might change in the future for tropical regions [10, 288]. Under some scenarios, UV-B radiation could increase by 3% in the tropics due to interactions between stratospheric ozone, climate and aerosols [8, 10]. This increase would further elevate the already high levels of UV-B radiation that occur naturally at low latitudes. At present, the consequences of these relatively modest percentage increases in UV-B radiation on crops or wild plants in this region are uncertain. Available evidence suggests that current levels of UV-B radiation in the tropics can alter the morphology (e.g. smaller leaves, reduced shoot height) and chemistry (e.g. increased flavonoid levels) of native, non-crop tropical plants, but that biomass production is rarely decreased in these species (e.g. [289]). By comparison, several field experiments have shown that certain varieties of temperate-zone crops (e.g. wheat and soybean) [290–292] show decreases in photosynthesis and yield when grown under ambient UV-B radiation in the tropics. These findings suggest that some important crop species grown in the tropics might be vulnerable to relatively small increases in UV-B radiation.

As noted above and in Sect. 4.1, climate change is shifting bioclimatic zones and this is allowing certain crops to be grown at higher elevations than was previously possible [293–298]. For some crop species originating from lower elevations, the more intense UV radiation at high elevations may exceed their tolerances to UV radiation with negative consequences for their physiology and growth [299]. As crop species are grown in these new habitats, they will also encounter new weeds, pests and pathogens, which may disrupt the structure and function of these agroecosystems [300, 301]. Differential effects of climate change on range shifts and phenology can also lead to spatial and/or temporal or seasonal mismatches between pollinators and their plant hosts [302, 303], posing additional risks to food security. Many of these high-elevation agroecosystems support community livelihoods and are important carbon sinks that help mitigate global warming. Thus, their risks from changes in climate and UV radiation are of particular concern.

### Effects of UV radiation on food quality

Laboratory and field studies have found significant effects of UV radiation on crop quality with regard to texture, flavour, appearance and nutritional content. It is now well-established that the concentrations of a wide array of natural plant chemicals are modified by UV radiation [304–307] and these changes in chemical composition can have positive and negative effects on food quality. There is abundant research demonstrating that exposure to modest levels of UV radiation can improve food quality by enhancing crop flavour [308], taste [309], colour [310], nutritional content [311–314], and pharmaceutical content [315–317] in various plants. Given that the intake of fruits and vegetables of many consumers is well below recommended levels [318], the higher nutritional content of crops exposed to UV radiation may generate long-term health benefits. For example, Keflie et al. [319] used solar UV-B radiation to increase vitamin D in oyster mushrooms, which may alleviate vitamin D deficiency in humans. Some have proposed legal regulation for UV treatment of foods, including mushrooms [320].

In some cases, exposure of crops to UV radiation can lead to a decrease in their nutritional value for humans and livestock. For example, some species of tropical grasses show increases in tannins when grown under experimentally elevated UV-B radiation, and this would imply a reduced palatability of forage for cattle [321, 322]. High levels of UV radiation may also increase amounts of other anti-nutritional compounds in plants, such as oxalates, which are generally associated with kidney problems [323]. At present, the full scope of UV-induced anti-nutritional compounds is not fully known nor is the identification of crops most at risk to these changes.

### Effects of UV radiation on plant interactions with pests and pathogens

The Food and Agriculture Organization of the United Nations (FAO) estimates that plant pests^2^ cause a 20–40% loss in global agricultural production per year, costing ca. $220 billion USD, with the impacts of invasive insect species adding another $70 billion USD [324]. It is expected that climate change, including ECEs, will increase the incidence and severity of pests and pathogens in some regions, as these organisms colonise new previously sub-optimal habitats along latitudinal and elevational gradients [325, 326]. The climate-induced parallel range shifts of plants with latitude or elevation into new habitats may constitute additional stress from plant pests (Sect. 4.1) [327]. Rising concentrations of CO_2_ and associated global warming together with regional increases in UV radiation may also act together to compromise food security through complex effects on plant pests and disease [328]. While our previous assessments have reported on UV-mediated increases in resistance to specific pests and pathogens [12, 38], we note that there is a need for more detailed studies on the interactive effects of UV radiation, CO_2_ and other climate change factors on plant interactions with pests and pathogens.

Exposure to UV radiation can confer increased resistance of certain crops to pests and diseases through changes in host physiology, morphology, and biochemistry. As noted in Sect. 3.1, UV radiation typically enhances the production of polyphenolic compounds, such as flavonoids. Some of these compounds enhance a plant’s defence against herbivores and pathogens (e.g. viral, fungal or bacterial) [239, 282, 329]. Disease and pest attack will also elicit the production of increased amounts of these polyphenolic compounds that can make the host plant unpalatable or toxic (Sect. 5.2) and/or protect the plant through their antioxidant properties (e.g. scavenging of free radicals). These effects on pests or pathogen attack are part of a wider network of interactive effects on plant physiology and morphology potentially altering the susceptibility of crops to these threats [330].

Chemical biocides are widely employed to manage pests and pathogens in crops (Sect. 5.4). However, several biocontrol agents against insect pests have been developed and used as alternatives to chemical pesticides. Of particular interest is a group of fungi that are parasitic on insects (entomopathogenic fungi). Entomopathogenic fungi kill insects by penetrating the outer protective cuticle layer of specific hosts with the help of proteases [331]. These fungi live naturally in soils but can be mass-produced for application to crops where they have been used against pests including spittlebugs and locusts, which affect crops such as maize, sugarcane and beans [331], as well as against various insect pests in rice [332]. However, many of the entomopathogenic fungi are strongly inhibited by UV radiation and temperatures above 30^0^ C, which affect their development and pathogenic function against certain insects. Therefore, these abiotic constraints are considered a major barrier to the use of entomopathogenic fungi in controlling insect pests [332–335]. However, the effect of solar UV-B radiation on these fungi remains to be confirmed through experiments where they are grown under realistic solar radiation conditions [336]. Such studies may also allow for selection of fungal biocontrol agents that are more tolerant to UV-B radiation and other climate factors, for use as biocontrol agents to safeguard economically important agricultural systems.

### Effects of UV radiation on agricultural biocides

The widespread application of biocides (herbicides and pesticides) for controlling or killing harmful organisms in agricultural field settings results in some accumulation of these chemicals in water, soil, and atmosphere, and may also result in residues in agricultural products. Given that biocides are designed to be bioactive, their adverse effects on non-target organisms and humans are of concern. Direct and indirect photodegradation by solar UV radiation can potentially reduce the environmental residence time of pesticides [19]. However, photochemical degradation can also reduce the functional effectiveness of biocides as crop protectants, which may lead to greater amounts being administered by growers [337, 338]. Direct photodegradation of biocides occurs when a chemical absorbs UV radiation, leading to its breakdown into various degradation products [339]. Indirect photodegradation involves the reaction of the biocide with reactive intermediates formed when natural photosensitisers (e.g. nitrate) absorb solar radiation [340]. Not all biocides are subject to direct photodegradation under solar radiation. For biocides with an action spectrum for direct photodegradation only in the UV-C region (wavelengths 100–280 nm) and not extending into the solar UV-B, only indirect photodegradation occurs under solar radiation.

In the field, the exposure of biocides to solar radiation depends on the manner in which they are applied to crops, as well as the specific characteristics of the crops, including age and canopy structure, which determines their exposure to solar radiation. These factors, together with the chemical composition of the pesticide formulation determine the extent to which they are photodegraded in the field. For example, the additive (co-formulation compound) benoxacor, which is used as a safener (i.e. a compound used in combination with herbicides to reduce negative effects on crops) of the herbicide metolachlor, accelerates the photodegradation of the active ingredient on soil surfaces, lessening its toxicity [341]. The extent to which biocides are photodegraded is also highly dependent on where the biocide residues occur. For example, the photodegradation rate of the herbicide imazethapyr is two orders of magnitude slower when applied to maize and soybean leaves than in aqueous solutions [342]. The leaves of aromatic herbs like thyme emit volatile organic compounds that can further affect the photodegradation of biocides deposited on their leaf surfaces, resulting in the formation of different photoproducts [341, 343–345]. Thus, the importance of direct vs. indirect UV-mediated photodegradation of biocides in the environment appears highly context dependent, and requires further research across a range of crops, environmental conditions and methods of application to clarify modes of action.

Climate change may be an additional factor impacting pesticide photodegradation on leaf surfaces. While photodegradation kinetics typically have a weak temperature dependence, pyrethroid insecticides applied onto spinach plants grown at 16–21ºC degraded up to 2 times slower than when plants were grown at lower temperatures (10–15 ºC), likely due to differences in the chemical composition of leaf wax [346].

As observed for other contaminants [e.g. 348,112] biocide photodegradation products can be more toxic than their parent compounds. For example, some breakdown products generated by UV-B radiation of the fungicide chlorothalonil and the insecticide imidacloprid on plant leaves are more toxic to fish than their parent compounds [344].

Functional nanopesticides are being developed using nano-emulsion technologies as an alternative to traditional pesticide applications [348–351]. Encapsulated pesticides in nano-carriers, such as polymers, nanoclays, and metal organic frameworks provide controlled-release kinetics and improved stability against environmental degradation by UV radiation. The use of encapsulated pesticides prevents undesirable pesticide losses and release into the environment that otherwise would cause ecological and health concerns [352]. The development of nano-biocides may contribute to more environmentally friendly and sustainable food production systems (Sect. 7), potentially protecting the integrity of biocides during their application on crops, while still facilitating subsequent degradation of their residues.

### Development and application of UV lighting systems in agriculture

Concerns over the effects of elevated UV-B radiation resulting from ozone depletion on food production stimulated considerable research into the effects of UV-B radiation on crops, and much of this early research focused mainly on the leaf-level physiology and shoot growth of traditional crop plants (e.g. soybean, rice, maize; [353]). More recently, studies have examined effects of UV-B radiation on plants of medicinal value, mushrooms and algae [354]. For example, mushrooms [355, 356] and certain microalgae [357] synthesise increased amounts of vitamin D after being exposed to UV-B radiation (Sect. 5.2) [357, 358]. In addition, more attention is being given to studying the effects of UV-B radiation on seeds, fruits, subterranean organs (e.g. roots and tubers), and on derived products, such as wine and olive oil [312, 359–362].

Results from these studies indicate that plants exposed to low or moderate levels of UV-B radiation in controlled environments (e.g. greenhouses, growth chambers) often have improved vigour, enhanced nutraceutical quality and are more resistant to pest and pathogens compared to plants that are grown in the absence of UV-B radiation, as typically occurs in commercial production glasshouses [281].

Other studies have shown how the application of UV-B radiation can modulate different physiological processes important for agriculture. These advances include, (1) accumulation of anthocyanins and other antioxidants in different coloured fruits, such as peach, apple, grapes, and blueberry [313, 362–365] (Sect. 5.2); (2) improving the tolerance of rice and tomato to low temperatures, salinity and drought [366, 367]; (3) the manipulation of flavonoid accumulation in vegetables [368]; (4) the production of smaller cucumber plants for targeted commercialisation [174]; (5) an increase in anticancer compounds in *Catharanthus roseus* [369] following treatment with a combination of hormones and UV radiation; and (6) extending the shelf-life of fruit by reducing the activity of enzymes involved in fruit rotting [370]. Also, the accumulation of bioactive compounds can be triggered more effectively by applying high UV-B radiation during short periods in specific developmental stages (frequently near harvest) rather than using UV-B radiation over longer periods. This approach has been successfully applied in kale and grapes [361, 371, 372]. These advances have been achieved by translating research that was conducted to better understand the effects of increased UV-B radiation resulting from ozone depletion into commercial practices to improve food quality and production (Sect. 7).

One of the more significant technological advances in plant UV research and horticulture has been the development and use of UV light-emitting diodes (LEDs). Increasingly, LED lighting systems are being used by growers before and after harvest to improve food value. LEDs are more energy-efficient and environmentally friendly than most traditional light sources used in horticulture (e.g. high-pressure sodium vapour or metal halide lamps), and by utilising LEDs that emit both in the UV and PAR regions the control of the spectral composition, intensity and exposure period can be attuned to the light requirements of specific plants and crops [373, 374]. However, at present, only UV-A LEDs have been widely adopted to stimulate the accumulation of desirable plant compounds [375]. There are also some examples of successful application of UV LEDs in reducing certain plant diseases [376] and increasing nutritional quality [375].

## Effects on biogeochemical cycles and climate feedbacks

Terrestrial ecosystems provide many valuable services, including the processing of dead organic material and the storage and recycling of essential nutrients. Both land vegetation and soils are also important carbon sinks that influence the concentrations of atmospheric CO_2_ and hence climate. Solar UV radiation affects carbon storage and atmospheric CO_2_ by influencing plant productivity [3], and the photodegradation of modern dead plant material (litter) and ancient organic matter preserved in permafrost soils [377, 378], which becomes exposed to solar radiation because of climate change-induced thawing [379–383] (Box 2). Changes in climate and UV radiation can further interact to alter the cycling of other elements (nitrogen being the most important) and the emissions of GHGs other than CO_2_, which can affect stratospheric ozone and climate. Below, we evaluate new findings that address the underlying mechanisms and climate consequences of the interactive effects of UV radiation and climate change on biogeochemical cycles.

### Photodegradation of plant litter

The decomposition of plant litter is a key biogeochemical process determining rates of nutrient cycling and energy flow in terrestrial ecosystems. This process affects vegetation productivity, carbon storage and soil fertility, and releases CO_2_ and other GHGs to the atmosphere [384]. Thus, decomposition of litter has important feedback effects to the climate system.

**Fig. 3:**
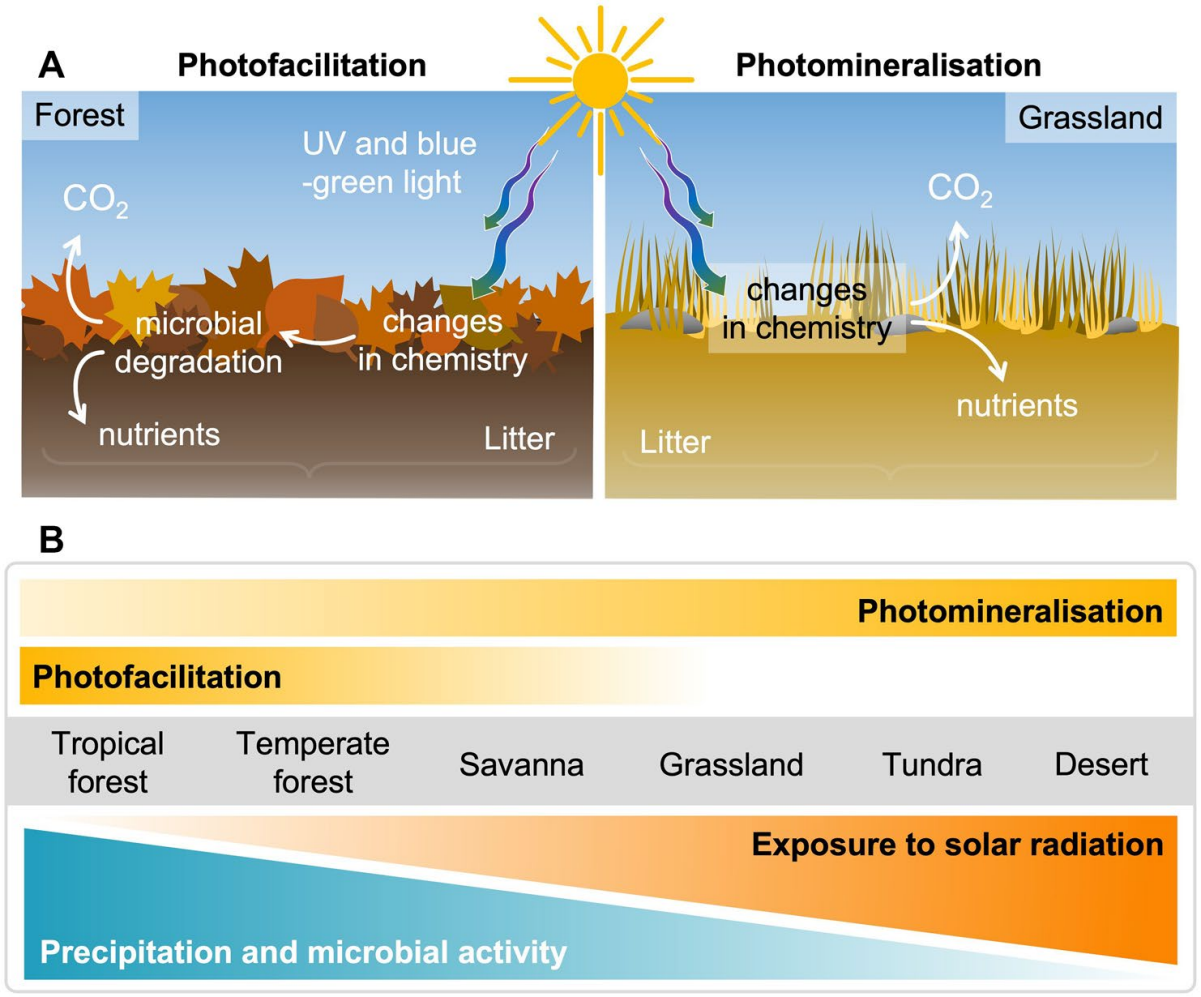
The relative importance of photomineralisation and photofacilitation in litter decomposition across terrestrial biomes and environments. Panel **A** illustrates the processes of photofacilitation and photomineralisation in the photodegradation of surface litter exposed to solar radiation (UV radiation and blue–green light) in representative wet (forest; greater photofaciliation) and dry (grassland; greater photomineralisation) ecosystems. Panel **B** shows relative changes in photofacilitation and photomineralisation across biomes and along gradients of moisture, microbial activity and exposure to solar radiation. Not shown in this figure is the potential leaching of non-volatile breakdown compounds resulting from photodegradation of litter that can occur when it rains, and possible negative direct effects of UV radiation on microbes

In general, the rate of litter decomposition is regulated by climatic factors (temperature and moisture) and the chemical composition of litter (primarily the amount of lignin and the ratio of carbon to nitrogen (C:N ratio) in the litter), which modifies the activity and composition of the decomposer organisms (fungi, bacteria and invertebrate decomposers). Exposure of litter to solar UV radiation and short-wavelength visible radiation (i.e. blue and green light), can cause the direct breakdown of lignin and other plant cell wall constituents forming non-volatile and volatile compounds (e.g. CO_2_ which is released to the atmosphere). This process is referred to as photochemical mineralisation or photomineralisation [384, 385] (Fig. 3A, right panel). Additionally, UV and short-wavelength visible radiation can also accelerate the breakdown of litter by changing its chemistry, making it more palatable to microbes and thereby enhancing microbial decomposition (Fig. 3A, left panel) [386–388]. Promotion of microbial activity can also occur by the photodegradation of waxy surfaces layers (i.e. leaf cuticle) that allows moisture to more readily penetrate litter [389]. These indirect effects of solar radiation on microbial decomposition are collectively referred to as photo-priming or photofacilitation [390, 391]. In some situations, solar UV radiation can negatively affect litter decomposition by altering the composition and activities of the decomposer community (not shown in Fig. 3) [392]. The overall effect of solar radiation on litter decomposition reflects the net effect of these three processes [390].

Among litter components, lignin has been identified as the most photoreactive due to its absorption in the UV and blue–green region of the solar spectrum [384, 391]. However, recent studies have found that cellulose and hemicellulose are even more susceptible to photodegradation than lignin [393, 394]. These discrepancies are an unresolved knowledge gap that could be addressed by identifying differences in the photodegradation action spectra for lignin, cellulose, and hemicellulose. The presence of polyphenolic compounds in plant litter (Sect. 3.1) decreases photodegradation under natural [395] and controlled laboratory conditions [396]. This result suggests that the accumulation of polyphenolic secondary metabolites in green leaves may persist during the early phase of litter decomposition and attenuate the penetration of UV-B radiation into litter. The surface area of litter exposed to solar radiation is also an important predictor of litter decomposition rate and carbon turnover [397–399].

Photodegradation of litter was initially thought to be important only in dryland ecosystems (e.g. deserts and grasslands) where low moisture and high temperatures often constrain the activities of decomposing microbes. Recent studies have established that photodegradation of litter is important not only in semi-arid [400–402] and arid [403–406] ecosystems but also in moist environments that support tropical [407], subtropical [408], temperate and boreal forests [392, 395, 396], alpine steppe [409], and marshes [410].

Calculations of the strength of the terrestrial carbon sink have typically excluded photodegradation of litter in mesic ecosystems (having moderate water supply) due to their high vegetation cover. However, recent field studies found that photodegradation of litter facilitates carbon cycling in canopy openings of temperate and tropical forests, even where understory solar radiation is relatively low [395, 396, 407, 411]. Exposure to the full solar spectrum, resulting from the formation of a forest gap, can increase litter photodegradation rates by up to 120% relative to shaded conditions across a wide diversity of plant species [395]. This number is considerably higher than that for photodegradation in semi-arid regions (60%) [412] or across several habitats or biomes (23%) [386], underscoring the importance of forest disturbance in mesic ecosystems. Exposure to solar radiation alters lignin structure of litter in the early stages of decomposition, promoting litter degradation via photofacilitation. This fact highlights the role of photofacilitation in mesic ecosystems, where higher water availability favours microbial decomposition compared to drylands [387, 413] (Fig. 3). On the other hand, relatively high UV radiation, which occurs during the time of the year when the forest canopy is leafless, may also have an inhibitory effect on microbial decomposers [396]. The seasonal consequences of these effects of UV radiation on understory microbes and overall ecosystem health and function remain unclear.

**Fig. 4:**
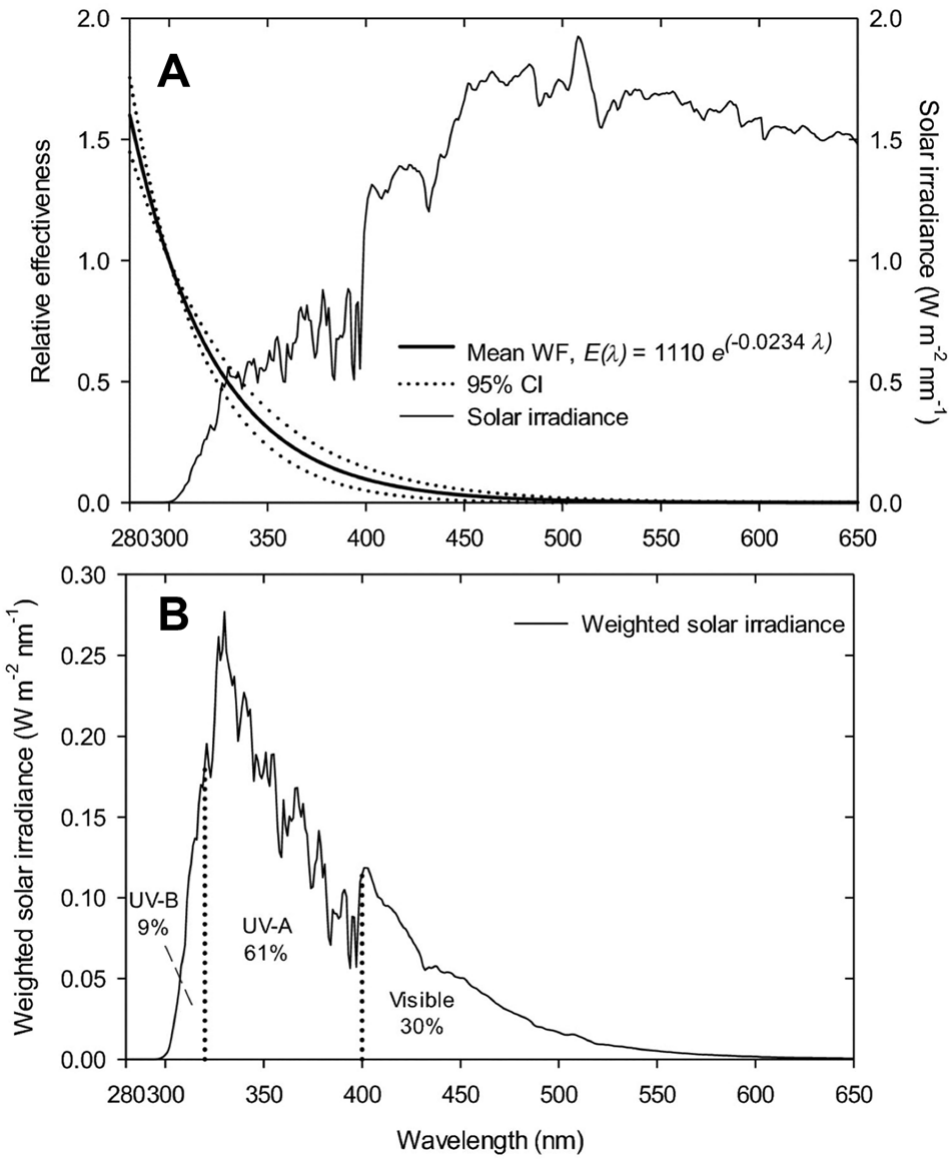
Action spectrum and weighted solar radiation for the photomineralisation of litter from plants in the North American Sonoran Desert. Panel **A** shows the mean weighting function/action spectrum for the photo-mineralisation of plant litter (heavy solid line; measured as CO_2_ loss), with 95% confidence interval (dotted line; CI), along with the average solar noon spectral irradiance over the time period of the study (thin solid line). Panel **B** shows solar radiation at solar noon weighted according to the action spectrum in Panel A, along with the total % effectiveness of the solar UV-B, UV-A and visible wavebands. Adapted from [404]

Recent studies have clarified the relative importance of the different wavelengths of solar radiation (i.e. UV-B, UV-A and blue–green) in driving photodegradation of litter and these findings have implications for the effects of ozone depletion on this process. A recent meta-analysis found that, globally, solar radiation increases litter mass loss by 15.3 (± 1.0)% relative to litter that has not been exposed to solar radiation [414]. The contribution of UV-B radiation was found to be significant only in specific environments, causing an 18% and 23% loss of litter mass in semi-arid regions and polar regions, respectively. The relatively limited importance of UV-B radiation in promoting loss of litter mass agrees with the results obtained with a new spectral weighting function for litter photomineralisation, which showed that UV-B and UV-A radiation, together with visible blue-green light, are responsible for 9%, 61% and 30%, respectively, of total photochemical (abiotic) carbon loss [404] (Fig. 4). Overall, these rather small effects of UV-B radiation suggests that litter photodegradation would be minimally affected by further changes in stratospheric ozone.

Photodegradation can also be influenced by changes in vegetation cover and soil erosion that result from changes in land use and climate, including ECEs (Sect. 2.2). The loss of forests and other natural or semi-natural vegetation cover due to agricultural practices increases photodegradation of surface litter [19, 397], such that deforestation and land clearing will accelerate release of carbon from the ecosystem and alter patterns of GHG emissions and nutrient cycling [395, 402, 415, 416]. In dryland ecosystems, litter position (e.g. at the soil surface vs. buried or covered in dust) is the predominant factor determining carbon loss from photodegradation [398, 417]. In contrast to forests, these dryland systems are experiencing an increase in woody plant cover as a result of changes in land use and climate and these vegetation shifts result in more shading of ground litter and increased soil erosion and deposition, which decrease litter photodegradation [418, 419]. Additional environmental changes such as increased nitrogen deposition and abandonment or less intensive use of agricultural land may slow litter decomposition through the attenuation of surface UV radiation by increased plant canopy development [409].

Rainfall is another factor affecting litter photodegradation. In an experiment performed in drylands, the addition of supplemental precipitation (simulating a 2.7 times increased rainfall) accelerated loss of litter mass by a factor of 2.6 under near-ambient solar radiation but had no effect if litter was not previously exposed to solar radiation [388]. This result suggests that photodegradation followed by leaching may be another significant mechanism of loss of litter mass in arid ecosystems [388, 420].

Collectively, these findings indicate that the overall effect of photodegradation on the decomposition of plant litter depends on environmental conditions (primarily moisture and temperature), litter quality, the degree of exposure of litter to solar radiation (as influenced by vegetation cover, litter position and degree of soil–litter mixing), and the solar spectral composition of radiation reaching the litter layer [12]. Given the relatively small contribution of UV-B radiation to loss of litter mass and photomineralisation, ongoing and projected changes in stratospheric ozone and their interaction with climate and land-use changes are likely to impact litter photodegradation mainly by modifying its exposure to total solar radiation [12, 19, 145].

### Photochemical release of nutrients from terrestrial ecosystems

Most studies of photodegradation of organic matter in terrestrial ecosystems have focussed on effects on carbon but, as demonstrated in aquatic ecosystems [111], UV radiation can also affect the storage and cycling of other elements, such as nitrogen and phosphorous. Even in understory environments, where the amount and spectral composition of solar radiation is greatly modified by canopy structure and phenology, UV-B radiation [395], UV-A radiation and blue light can promote the conversion of organic nitrogen into inorganic compounds (nitrogen mineralisation) [411, 415].

A recent meta-analysis of litter degradation studies found that the amount of UV radiation received affected the timing of nitrogen and phosphorous loss compared to that of carbon [413] due to differences in the relative contribution of microbial *vs.* photochemical degradation. Under reduced UV radiation, nutrient mineralisation was slow and poorly correlated with overall loss of litter mass, whereas, under increased UV radiation, phosphorous and nitrogen mineralisation was rapid and correlated with carbon mineralisation. These results suggest that microbial processes dominate nutrient cycling under low levels of UV radiation, while abiotic processes, which are characterised by a simultaneous release of nutrients and carbon, are more important at higher UV irradiances. Thus, under conditions of high UV irradiation the nutrients in litter may be made more rapidly available to plants, potentially reducing competition for nutrients between plants and microbes. These effects could play a significant role in ecosystem functioning but have not yet been thoroughly studied. The release of mineral forms of nitrogen is also likely to produce volatile nitrogen compounds including nitrous oxide (N_2_O), which is both a powerful GHG and ODS [19]. Given the obvious implications for climate and stratospheric ozone, the effect of UV radiation on N_2_O emissions by litter remains a critical knowledge gap to be addressed in future studies.
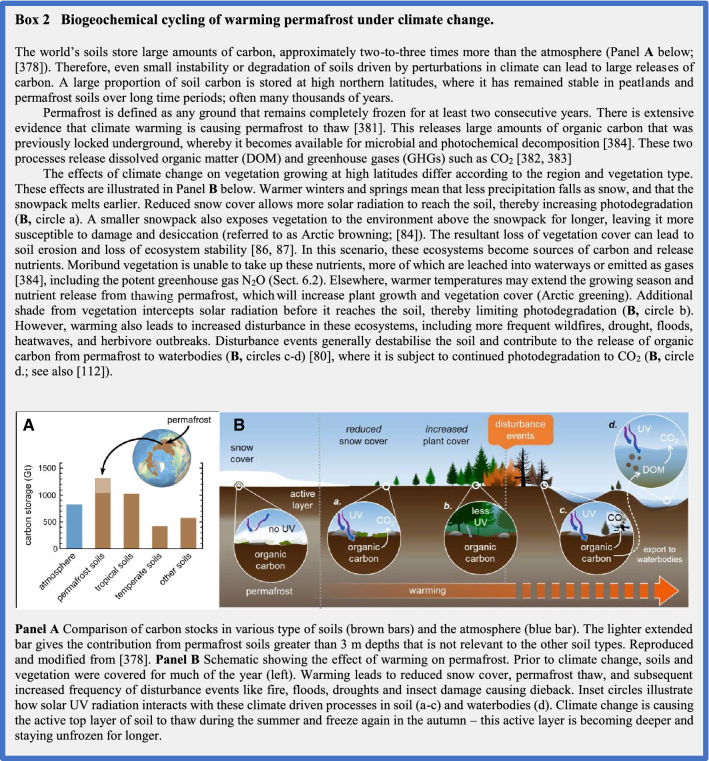


### Methane emissions, UV radiation and plants

Methane (CH_4_) is a potent greenhouse gas, such that relatively small changes in its emissions can make a significant contribution to climate change [421]. In addition to anthropogenic emissions, methane is released naturally by terrestrial ecosystems, particularly wetlands [422, 423]. At present, solar UV radiation is not considered an important driver of methane emission from terrestrial ecosystems [9, 111, 424]. However, there is concern that climate changes associated with stratospheric ozone depletion at high latitudes (tundra and taiga ecosystems in the Northern Hemisphere and peatlands or wetlands in the Southern Hemisphere) may enhance methane emissions [19].

Plants often serve as conduits of methane produced by bacteria in damp soils [425]. They also contribute to methane emissions through photochemical mineralisation of pectin, waxes, and lignin by UV-B radiation, although this effect is deemed rather small [426]. Methane emission from plants is accelerated by interaction with other stressors such as herbivore damage and high temperatures [427]. Controlled experiments with Scots pine and Norway spruce under ambient conditions in Finland found a positive relationship between methane emissions and solar radiation, which was steeper at warmer temperatures [428]. Even then, in most habitats, direct emission from plants through photodegradation of pectin [429] is considered only a minor contributor to global terrestrial methane emissions [421, 428].

Methane emission from plants also occurs through microbial methane production in the heartwood of trees (reviewed by [421, 424, 430]). From there, methane can be released to the atmosphere by passing through the bark or through the plant’s vascular system. This process is currently thought to be the main avenue of plant methane emissions in non-wetland environments, and it is modulated by the moisture and phenolic content of heartwood rather than by UV radiation [431]. Reactive oxygen species (ROS), which are produced in all organisms and can be enhanced by oxidative stress, also take part in reactions that can release methane. Additional research is required to at the global scale to provide for a more complete understanding of the effects of climate and UV radiation on terrestrial methane emissions [421, 424, 427, 432].

### Interactions of UV radiation with fire-derived carbon

Forest fires are increasing in severity and frequency and will become even more prevalent as the climate continues to change [20]. Boreal forests are particularly vulnerable to fires as extreme warming is expected in this region [433–439] close to the Arctic circle. Forest fires directly contribute to climate change by releasing GHGs such as CO_2_, methane, and nitrous oxide [15, 434]. Wildfires also provide an important pathway for opening soil surfaces to UV irradiation, leading to enhanced photodegradation of organic matter with consequent release of CO_2_ (Sect. 6.1; Box 2). Due to the incomplete combustion of wood and other biomass, fires convert a substantial fraction of vegetation into burnt biomass, termed charcoal or pyrogenic carbon (PyC) [436]. Recent estimates indicate that *ca*. 256 Tg carbon (Tg_C_) yr^−1^ (range = 196–340 Tg_C_ yr^−1^; 1 teragram = 10^12^ g) of biomass were converted into pyrogenic carbon between 1997 and 2016 [440]. During rainfall events following a wildfire, ash and pyrogenic carbon (estimated up to 203 Tg_C_ yr^−1^ in a modelling study [441]) reach nearby watersheds, resulting in increased loads of organic carbon, nutrients, and metals [442, 443]. The impact of wildfires on surrounding water bodies can last for years, affecting biogeochemical processes and drinking water quality [442, 444]. In addition, fire-derived aerosols can temporarily reduce incident UV radiation reaching the Earth’s surface [144] and slow down UV-driven chemical processes in the troposphere [15].

Pyrogenic carbon includes a broad suite of chemicals such as anhydrous sugars, condensed aromatics (often named black carbon), and graphitic carbon [440]. The specific chemical composition of PyC depends on biomass type and charring temperature, and this composition affects its solubility, bioavailability, and photoreactivity [440, 443, 445, 446]. Adding to previous findings [440], recent studies confirmed that black carbon is the most photoreactive fraction of PyC [447] and that microbial mineralisation of PyC can be enhanced by prior exposure to UV radiation (i.e. photofacilitation) [443, 446], similar to plant litter (Sect. 6.1) and dissolved organic matter in water [111].

## Sustainability and the Montreal Protocol

**Fig. 5:**
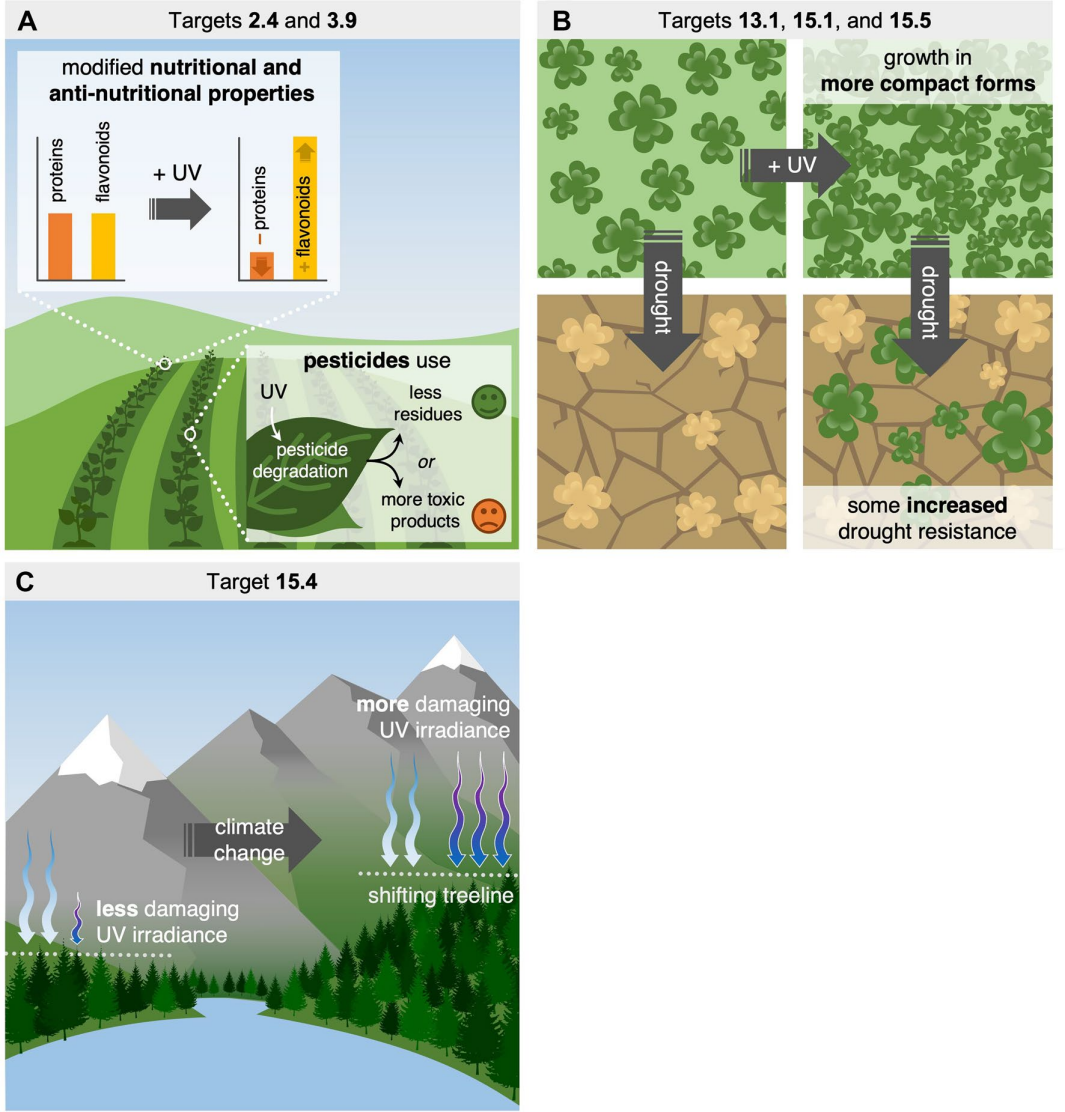
Pictorial representation of how the Montreal Protocol and its Amendments align with several Sustainable Development Goals (SDG) and their targets. Panel **A** shows SDGs **2.**4 (*Sustainable food production and resilient agricultural practices*) and **3.**9 (Deaths and illnesses from hazardous chemicals and soil pollution and contamination). Panels **B** and **C** show SDGs 13.1 (Strengthen resilience and adaptive capacity to climate related disasters; centre panel) and 15.1, 15.4, 15.5 (Protect, restore and promote sustainable use of terrestrial ecosystems, sustainably manage forests, combat desertification, and halt and reverse land degradation and halt biodiversity loss)

By protecting the stratospheric ozone layer and mitigating some of the effects of climate change, the Montreal Protocol and its Amendments are assisting in the implementation of several of the United Nations Sustainable Development Goals (SDGs). Many findings in our Quadrennial Assessment address SDGs and specific targets that are relevant to agriculture (*SDG 2: Zero hunger*) and terrestrial ecosystems (*SDG 15: Life on land*) (Fig. 5). Other relevant contributions of the Montreal Protocol are related to pollution and contamination (SDG 3: *Good health and well-being*), and climate change (*SGD 13: Climate action*). Specific SDG targets addressed by our findings are described below.

### SDG 2: Zero hunger

While small increases in solar UV-B radiation do not appear to pose a threat to crop yield, extreme increases in UV-B radiation, as would have occurred without the Montreal Protocol, would likely have significantly decreased agricultural productivity (Sect. 5) and jeopardised SDG 2 and particularly **2.**4 (*Sustainable food production and resilient agricultural practices*). Several studies have shown that moderate UV radiation can alter the chemical composition of food and medicinal plants (Fig. 5a). In most cases, UV radiation increases the nutritional profile of some crop species (e.g. by increasing the concentration of certain antioxidants, e.g. flavonoids), with potential long-term positive outcomes for human health (Sect. 5.2). The latter finding has motivated the development of agricultural practices (e.g. UV-transparent greenhouse coverings and UV-emitting LEDs) that exploit low and medium levels of UV-B radiation to enhance the nutraceutical properties of crops (Sect. 5.5). These practices can be directly deployed in both developed and developing countries to obtain food with an improved nutritional profile for increased food security.

### SDG 3: Good health and well-being

Plants exposed to modest levels of UV radiation often display some increase in resistance to pests and pathogens (Sect. 5.3), which could lead to reduced use of pesticides. However, solar UV radiation also degrades certain pesticides (Fig. 5a). This may lead to increased application of pesticides (Sect. 5.4), which could increase the risk of exposure of workers and consumers to these chemicals as well as adding to soil pollution and contamination (SDG **3.**9). The net result on pesticide use likely depends on many factors, including changes in UV irradiance, cropping system, and types of pesticides.

Insert Fig,

### SDG 13: Climate action

Modelling studies indicate that the Montreal Protocol and its Amendments have played a critical role in protecting global carbon sequestration by terrestrial vegetation, which has, in turn, slowed the build-up of CO_2_ in the atmosphere and reduced a certain amount of global warming (Box 1). Also, exposure of plants to modest levels of UV radiation, that would not have continued to occur without the Montreal Protocol, can improve their tolerances to drought (Fig. 5b) and enhance resistance to pests and pathogens, thereby making crops and natural ecosystems more resilient to climate change (Sect.  2.2, 3.3; SDG* Target 13.1: Improve resilience to climate change*). Finally, this Assessment prepared for the Parties to the Montreal Protocol and as a scientific publication contributes to *SDG Target 13.3* (*Improve education, awareness-raising and human and institutional capacity on climate change mitigation, adaptation, impact reduction and early warning*).

### SDG 15: Life on land

Increasing temperatures due to climate change are shifting the distribution ranges of plants and animals to higher elevations and latitudes, which changes their exposure to solar UV irradiation (Fig. 5c; Sect. 4.1). Some modelling studies suggest that UV radiation can be important in influencing the distribution shifts in plants (Sect. 4.2), which have the potential to negatively impact biodiversity (*SDG Target 15.1: Conservation of terrestrial ecosystems*). For mountain ecosystems, the shift to higher altitudes is often more pronounced for invasive species, which then occupy ecological niches of endemic alpine species with negative outcomes for biodiversity (*SDG Target 15.4: Conservation of mountain ecosystems*).

### SDG 17: Partnership

Monitoring of the stratospheric ozone layer and its interactions with climate change are key to understanding the effects of UV radiation on terrestrial ecosystems, therefore the assessment of how species respond to this climatic pressure represents a challenge imposed on all countries. Partnerships between countries in Northern and Southern Hemispheres have been facilitated by the Montreal Protocol, which has stimulated technology transfer and innovation on the effects of UV radiation on plants and animals among scientific communities worldwide (*SDG targets 17.6, 17.7 and 17.8: North–South cooperation to access science, technology and innovation; Promote development, transfer and dissemination of environmentally sound technologies; and Science, technology and innovation capacity-building mechanisms for least developed countries)*. This partnership has facilitated international support for data acquisition and sharing on the stratospheric ozone layer and the effect of UV radiation on terrestrial ecosystems (*SDG target 17.9*: *Enhance international capacity-building to achieve SDGs*). This has assisted least developed countries to have first-hand information for the implementation of environmental policies towards the achievement of SDGs (SDG target 17.14: *Enhance policy coherence for sustainable development*).

## Gaps in knowledge

In this assessment, we have identified several important knowledge gaps. These include:

- **Additional well-designed field studies are needed on all the topics addressed here to increase the confidence in our assessment.** It is well-established that the responses of plants and other organisms to UV radiation are heavily dependent on other wavelengths of solar radiation as well as environmental factors such as temperature and moisture availability. There is also large inter- and intraspecific variation in sensitivity to UV radiation. Thus, the assessment of the effects of changes in solar UV radiation, stratospheric ozone and climate requires research conducted on a variety of species under natural, field conditions. However, studies conducted under controlled environmental conditions (e.g. growth chambers and glasshouses) can provide important insights into the mechanisms of effects of UV radiation. In our assessment we have included certain studies carried out under controlled conditions when the results appear plausible and/or are useful for increasing awareness of potential effects and outcomes, but more field studies are clearly needed to reduce many of the uncertainties identified in this assessment.
- **Research into the impacts of Solar Radiation Management (SRM), such as Stratospheric Aerosol Injection (SAI), is needed to keep pace with policy-makers’ interest in these technologies.** This is of particular concern, given that the existing evidence might suggest that impacts on terrestrial ecosystems of adopting SAI, and in particular any eventual termination or interruption of SAI following its adoption, are likely to be considerable, persistent and in some cases irreversible [24, 26–28, 448]. Importantly, some models of the effects of SRM on primary productivity by terrestrial ecosystems only draw on estimates derived from relatively simple and short-term calculations of changing canopy-level light-use efficiency under SAI scenarios. Experimental evidence of the relative importance of short-term responses *vs* the long-term acclimation of photosynthesis to the changes in spectral composition and irradiance brought by SAI have yet to be assessed through controlled experiments. Thus, we are not in a position to confidently assess the effects of SAI on ecosystem-level carbon assimilation at this time.
- **Experimental studies are needed to verify findings from modelling studies aimed at quantifying the environmental consequences of extreme levels of solar UV-B radiation, as would have occurred with uncontrolled emissions of ODS. **While these modelling studies are powerful approaches to understanding the benefits of the Montreal Protocol, and assessing the risks of future changes in the stratospheric ozone layer [e.g. 3], they rely on several assumptions that can lead to large uncertainties in the findings. As experimental studies on organisms exposed to these extreme amounts of UV radiation are lacking, it is often assumed that the effects of UV-B radiation on growth, productivity and reproduction observed under current UV radiation can be linearly extrapolated to higher amounts of UV radiation. This assumption is likely unrealistic, especially for the more extreme ozone depletion scenarios that would have eventually occurred without the Montreal Protocol. In addition, little is known about how the photomorphogenic responses of plants, which are driven by photoreceptors such as UVR8, are affected by extreme levels of UV-B radiation, or about the levels of UV irradiation where damage by the UV-B waveband supersedes the regulatory, photomorphogenic effects.
- **There is a critical need to develop action spectra for plants and other organisms, which more accurately describe biological responses to the different wavelengths of UV radiation under the full solar spectrum. **Action spectra are fundamental to interpreting biological responses to changes in UV radiation that occur with stratospheric ozone depletion and they also serve as spectral weighting functions in both laboratory and field experiments [449]. Large uncertainties in assessing the effects of ozone depletion can occur if inappropriate action spectra are used [450].
- **The establishment of long-term biomonitoring studies would improve our ability to assess how organisms and ecosystems will respond to the ongoing changes in UV radiation and climate. **Changes in UV radiation and climate, especially extreme climate events and combined extreme events (e.g. wildfires), pose significant risks to the health, stability, and biodiversity of terrestrial ecosystems, but little experimental or modelling data exist to quantify these effects. These studies are critically needed for organisms and ecosystems in polar regions, the tropics and high-elevation mountains.
- **The establishment of a global UV radiation biomonitoring network using material from selected organisms (from pollen to plants and animals) could further increase our knowledge and reduce uncertainties on the use of biological proxies for solar UV radiation. **Certain plant material and tissues, such as herbarium specimens and pollen in sediment cores, have the potential to serve as proxies for reconstructing past UV radiation environments on Earth, but presently there are large uncertainties associated with these techniques.
- **Studies are needed to characterise a wider array of interactive effects to adequately assess the consequences and map potential mitigation options of ongoing changes in solar UV radiation together with other contemporary environmental changes. **Advances have been made in understanding how UV radiation interacts with other climate change factors (e.g. UV radiation and drought) to affect the growth and physiology of plants [451] but studies need to be expanded to include multiple interactive factors (e.g. UV radiation, temperature, drought, CO_2_ concentrations).
- **There is a need for additional biomedical research examining how UV radiation-induced changes in plant secondary metabolites affects dietary availability of metabolites, and the impacts of these changes on food quality and the epidemiology of human diseases. **Evidence continues to mount showing that exposure of plants to UV radiation alters their secondary chemistry and nutritional quality. But how these changes affect human health is largely unknown. This knowledge gap needs to be addressed to gain a fuller understanding of climate change-associated effects of UV radiation and their consequences for consumers, as well as the development of more sustainable agricultural practices (Sect. 5.5 and 7).
- **Research is needed to better understand the effects of UV-B radiation on animals. In comparison to terrestrial plants and ecosystems, there are far fewer studies on the effects of UV-B radiation on animals. **While there are some similarities in experimental approaches used to study plant and animal responses to UV-B radiation (e.g. providing different UV-B radiation treatments using UV-emitting lamps) there are also some important differences that often limit the applicability of UV radiation research on animals. For example, plant research typically uses a filter material (e.g. cellulose diacetate) to remove the short-wavelength UV radiation that is present in lamps but not in solar radiation [336]. Most plant research has also taken into account the effects of different UV wavelengths using action spectra as biological spectral weighting functions in designing and interpreting experiments using UV radiation produced by lamps [336]. Not all studies of the responses of terrestrial animals, including insects and other invertebrates, adopt these approaches. In our assessment, these experimental deficiencies represent a significant limit in placing current understanding of invertebrate responses, mostly obtained using UV-emitting lamps, in the context of variation in solar UV-B radiation in the field.
- **Despite recent advances in understanding the ecological significance of photodegradation in the decomposition of plant litter, further research is needed to refine our mechanistic understanding of this process and assess its importance in the cycling of carbon and other nutrients, and feedbacks to the climate system. **Findings since our last Quadrennial Assessment have revealed that photodegradation of plant litter is not only important in drylands, but across all terrestrial ecosystems. These findings explain, in part, why traditional biogeochemical models of litter decomposition that do not include photodegradation are often inadequate in reproducing measured mass and carbon losses [401, 452, 453]. Despite this general finding, many knowledge gaps remain, notably the quantification of the relative importance of photomineralisation *vs* photofacilitation in both dry and mesic environments, and whether the spectral weighting function derived from studies in drylands also applies to mesic ecosystems. Nutrient cycling has also been much less studied than carbon cycling, and particularly how changes in nitrogen cycling caused by UV irradiance could feedback on climate change and stratospheric ozone depletion. Open questions also remain concerning the underlying chemistry controlling litter photomineralisation and the role of UV radiation in driving GHG emissions from the thawing of permafrost. Reducing these uncertainties would improve our ability to assess how changes in UV radiation and climate will impact carbon cycling and feedbacks to the climate system.

## Conclusions

The findings presented in this Quadrennial Assessment indicate that changes in stratospheric ozone, UV radiation and climate can interact in various ways to modify terrestrial ecosystems and biogeochemical cycles. While exposure to solar UV radiation, and in particular the short-wavelength UV-B radiation, has the potential to cause deleterious effects on plants, animals, and microorganisms, most species have evolved mechanisms to tolerate or avoid harmful solar UV radiation at the Earth’s surface within the range experienced without significant ozone depletion. The extreme UV irradiances that would have occurred without the Montreal Protocol (i.e. “World-Avoided” scenarios) would likely have exceeded these tolerance limits and greatly reduced the productivity and biodiversity of terrestrial ecosystems. These conditions would also have driven increased photodegradation of organic matter and nutrient cycling, which would have increased emission of GHGs, including nitrous oxide, an ozone-depleting and greenhouse gas. Our findings further indicate that, in some cases, moderate levels of solar UV radiation (i.e. ambient UV irradiances without appreciable ozone depletion) can have some positive effects on organisms and the environment (e.g. improved food quality, enhanced plant defence against pests, improved plant vigour and resistance to other abiotic stresses, and the photodegradation of pesticides). Maintaining these beneficial effects of moderate UV radiation would have been impossible without the Montreal Protocol. Thus, the Montreal Protocol and its Amendments have played, and continue to play, a vital role in maintaining healthy, diverse ecosystems on land that can sustain life on Earth. The Montreal Protocol and its Kigali Amendment are also directly and indirectly protecting the Earth’s climate and mitigating some of the negative consequences of climate change by limiting the emissions of GHGs and protecting the carbon sequestration potential of vegetation and the terrestrial carbon pool [3, 5].

Since our last full assessment [12], there have been additional extreme weather events (e.g. heat waves, droughts, and hurricanes) and events resulting from a combination of weather extremes and other drivers (e.g. wildfires) that have all contributed to the disruption and destabilisation of terrestrial ecosystems. These have been particularly pronounced in polar regions where anomalies in stratospheric ozone and ozone-driven climate change have occurred in the last three years [10]. Ozone depletion over Antarctica in certain years has coincided with early summer and has likely resulted in greater exposure to UV radiation of animals, plants and microbes. These, and other extreme events (as outlined in Sect. 2.2), are expected to increase in frequency and intensity in the future because of climate change [20]. Together with other aspects of climate change, these extreme events will likely alter the UV radiation received by terrestrial organisms to a greater degree than the expected changes in the stratospheric ozone layer—assuming continued and full compliance with the Montreal Protocol. While understanding of the mechanisms of these UV-climate interactions is improving, the scale of their effects in terrestrial ecosystems remain poorly defined at present. Nonetheless, our findings indicate that the Montreal Protocol and its Amendments continue to make valuable contributions towards mitigating some of the negative environmental consequences of climate change as well as addressing several of the SDG targets established in the United Nations 2030 Agenda for Sustainable Development.

## Data Availability

All data generated or analysed are included in this article.

## Footnotes

1 An extreme climate event has been defined as “an episode or occurrence in which a statistically rare or unusual climatic period alters ecosystem structure and ⁄or function well outside the bounds of what is considered typical or normal variability” [112]; or similarly, according to the IPCC, “if the value of a variable exceeds (or lies below) a threshold” that is exceeded [20]. Compound extreme events are the “combination of multiple drivers and/or hazards that contribute to societal or environmental risk.” An example of a compound extreme event would be fire weather conditions which are the combination of hot, dry, and windy conditions [20].

2 A pest in this context is “any species, strain or biotype of plant, animal or pathogenic agent injurious to the plants or plant products”, as per the definition in the International Standard for Phytosanitary Measures No. 5 (ISPM) adopted by the Commission on Phytosanitary Measures of the International Plant Protection Convention.
